# Using enactive robotics to think outside of the problem-solving box: How sensorimotor contingencies constrain the forms of emergent autononomous habits

**DOI:** 10.3389/fnbot.2022.847054

**Published:** 2022-12-21

**Authors:** Matthew D. Egbert, Xabier E. Barandiaran

**Affiliations:** ^1^School of Computer Science, University of Auckland, Auckland, New Zealand; ^2^Te Ao Mārama, University of Auckland, Auckland, New Zealand; ^3^IAS-Research Center for Life, Mind, and Society, Department of Philosophy, University of the Basque Country (UPV/EHU), Bilbao, Spain

**Keywords:** enactive robotics, sensorimotor contingencies, problem-solving, habits, IDSM, sensorimotor autonomy

## Abstract

We suggest that the influence of biology in ‘biologically inspired robotics’ can be embraced at a deeper level than is typical, if we adopt an enactive approach that moves the focus of interest from how problems are solved to how problems emerge in the first place. In addition to being inspired by mechanisms found in natural systems or by evolutionary design principles directed at solving problems posited by the environment, we can take inspiration from the precarious, self-maintaining organization of living systems to investigate forms of cognition that are also precarious and self-maintaining and that thus also, like life, have their own problems that must be be addressed if they are to persist. In this vein, we use a simulation to explore precarious, self-reinforcing sensorimotor habits as a building block for a robot's behavior. Our simulations of simple robots controlled by an Iterative Deformable Sensorimotor Medium demonstrate the spontaneous emergence of different habits, their re-enactment and the organization of an ecology of habits within each agent. The form of the emergent habits is constrained by the sensory modality of the robot such that habits formed under one modality (vision) are more similar to each other than they are to habits formed under another (audition). We discuss these results in the wider context of: (a) enactive approaches to life and mind, (b) sensorimotor contingency theory, (c) adaptationist vs. structuralist explanations in biology, and (d) the limits of functionalist problem-solving approaches to (artificial) intelligence.

## 1. Introduction

Artificial Intelligence and the scientific approach to mind (what is known as *cognitive science*) was born (or rather raised) as a problem solving discipline (Newell et al., 1958; Putnam, 1965; Fodor, 1968). Deprived of life, the machine metaphor was one of symbol manipulation and rationality (deductive, inferential, heuristic or otherwise). The unprecedented potential of Universal Turing Machines (computers) was the driving metaphor to study the mind. The software, the mind, was the problem solving method, the hardware, the brain, its implementation.

Alternative conceptions of the mind were available at the origins of Artificial Intelligence and Robotics (Grey Walter, 1950; Ashby, 1952) but the rapid success of computer science left them aside. Over time, the limitations of the problem-solving centered computational theory of the mind became apparent and the biologically inspired, embodied and later enactive conceptions of the mind gained momentum. We are now immersed in a mesh of hybrid architectures, applied to a wide range of practices, from industrial to scientific modeling applications, and a new summer of Artificial Intelligence is rising, with robotics as a major container of social and technological expectations.

There are good reasons for why problem-solving attracts so much attention from researchers, but it is pertinent to ask: what aspects of minds are omitted or obscured by the problem-solving focused perspective? what can life teach us about what intelligence holds before and beyond problem-solving? and even when problem solving is addressed …how is it that natural agents have and become concerned by *their own* problems?

This paper has two goals. The first is to argue that by abandoning problem solving (or at least putting it down for a time), other useful explanatory targets and ways to explain minds are given space to emerge.

The second, more specific goal is a case in point: we use simulated robots to show how sensorimotor contingencies influence the formation of self-maintaining patterns of sensorimotor activity “habits” in regular ways that depend upon sensory modality. By de-emphasizing problem solving, we are able to take a fresh look at the relationship between sensory modalities, sensorimotor contingencies and habitual behaviors. But to explain these results, we first need to provide more context.

The paper proceeds as follows. The next section explains what we mean by “problem-solving,” why it has been a popular target within the cognitive sciences, and what we see as the primary disadvantage of excessive attention being given to the topic. We then explore the intimate relationship between robotics and biologically inspired and embodied problem-solving paradigms. Section 2.3 introduces the enactivist concept of autonomy, providing an alternative framework for developing Sensorimotor Contingency Theory outside of the problem-solving approach. The remainder of the paper presents and analyzes a simulation model that is used to explain: (i) that robots must first have their own problems instead of solving those posited by external observers; (ii) that, in doing so, they must assert a way of life whose structure and form must be taken as the object of study. We finally discuss some of the larger implications of our enactive approach in connection with wider theories of biological explanation and inspiration.

## 2. From problem solving to enactive robot

### 2.1. Problem-solving in minds and machines

We use the term “problem-solving” to refer, in a broad and inclusive manner, to the kinds of things that we associate with being capable or clever. Nowadays, for many, “the ability to solve problems is not just an aspect or feature of intelligence—it is the essence of intelligence” (Hambrick et al., 2020, p.553). It is certainly not a new idea. The very birth of Artificial Intelligence owes much to it (Newell et al., 1958). As Newell and Simon later stated: “Since ability to solve problems is generally taken as a prime indicator that a system has intelligence, it is natural that much of the history of artificial intelligence is taken up with attempts to build and understand problem-solving systems” (Newell and Simon, 1976, p. 120). The task of artificial intelligence was thus to devise potential solution-generators and to design tests that could evaluate them. This assumed that the problem space was well fixed so that solutions could both be evaluated and generated. Decades later, Artificial Intelligence handbooks still devote their first central sections to problem solving (e.g., see part II of Norvig and Russell, 2021, 4th edition).

A problem is a context in which behaviors can be evaluated according to a norm of success at “solving” the problem. Problems vary from being trivial to challenging to impossible as the proportion of behaviors that are good (out of all possible behaviors) shrinks. They include “high-level” human problem-solving, such as the skills that are taught in schools or universities, as well as embodied problems such as balancing on two feet or swimming efficiently. For a system to be evaluated in terms of problem-solving, one must first have the specification of the context and of a normative evaluation so that behaviors within that context can be compared as more or less successful. Defined as such, just about anything can be seen as a problem solver. A bottle lid solves the problem of preventing spills; a car's differential solves the problem of distributing force effectively to its wheels; a computer program solves the problem of beating a human at chess.

Herein lies both the advantage and the disadvantage of placing problem solving at the center of the cognitive sciences: *almost anything can be evaluated in terms of its problem solving ability*. On the one hand this is a great boon. Quantifying problem solving ability is relatively straight-forward and the ability to quantify how effective a system is at solving one or more problems facilitates technoscientific progress where measurements can play an important role in defining progress. This is apparent in artificial intelligence (AI) research where benchmarks such as chess and other games (Canaan et al., 2019), hand-writing recognition (e.g., Graves and Schmidhuber, 2009), image classification, speech recognition, etc. (e.g., MLPerf benchmarks MLPerf, 2021), are used to quantify progress. Problem-solving similarly provides metrics for studying the minds in psychology and neuroscience, where problem-solving related notion of ‘tasks’ (e.g., the Simon task) are used to structure human activity and performance related metrics such as reaction speed or error rate are seen as providing key insights into how our minds operate (Simon and Wolf, 1963).

The ease of measuring problem-solving ability sometimes leads to it (problem-solving ability) becoming the *explanandum*—the thing we strive to understand. This is seen in research questions like: *How do people recognize faces so well? How do babies come to understand the motives of other people? How do we play chess? How can we make a safe self-driving car?* etc. Problem-solving also sometimes becomes the *explananda*—the terms in which we explain what minds are, how they work, or what they do. Evolutionary psychology (Pinker, 1997; Buss, 1998), for example, emphasizes the evolutionary advantage of problem-solving ability and in this context, explanations that are provided in terms of problem-solving ability are seen as complete, as evolution can be invoked to explain why such mechanisms exist. The evolutionary advantage of having a mind is in its contribution to problem solving and therefore, minds are best understood as problem solving machines.

However, it has been argued that: “The essence of intelligence is to act appropriately when there is no simple pre-definition of the problem or the space of states in which to search for a solution. Rational search within a problem space is not possible until the space itself has been created, and is useful only to the extent that the formal structure corresponds effectively to the situation” (Winograd and Flores, 1987, p.98). Thus, even from a problem solving perspective, intelligence is not really the capacity to solve a problem but to bring a situation into a fabricated frame where it can be treated as a problem to be solved.

Moreover, the problem with excessive focus upon problem-solving is that there are other unique and important features of minds that are worthy of study—features that may only indirectly relate to problem-solving ability or perhaps not at all. The problem, in a nutshell, is the conflation of (i) “problem-solving ability” with (ii) all of the other phenomena associated with “being a mindful body.”

The mainstream computational functionalist approach to the mind (Putnam, 1965; Fodor, 1968) doesn't really help much addressing what mindful bodies are beyond problem-solving devices. For Putnam, the very definition of the mental is always in reference to a Turing machine table that works out rational transitions (e.g., computing and storing preferences over a utility function or solving problems in problem representation space). Deviations from this rationality are treated as pathological. All human mental life is, according to Putnam, not perfectly normal, thus relatively pathological. Putnam acknowledges “our model is an overly simple and overly rationalistic one in a number of respects. However, it would be easy, in principle, although perhaps impossible in practice, to complicate our model in all these respects—to make the model dynamical, to allow for irrationalities in preference, to allow for irrationalities in the inductive logic of the machine, to allow for deviations from the rule: maximize the estimated utility. But I do not believe that any of these complications would affect the philosophical conclusions reached in this paper” (Putnam, 1965, p. 43).

Deviations from the abstract rational rule are pathological. Explanation lies on the pure domain of abstract problem solving, the deviations make it all more complicated (as if dirt in the form of a set of exceptions where to be added to the pure explanation) but change fundamentally nothing. As we are about to see, embodied approaches, and particularly enactivism, bring these “pathological” expressions to the center of the explanation (of which rational thinking is the complicated achievement) turning it into the core constitution of mind. Biologically inspired robotics has a lot to contribute in this direction.

### 2.2. From biologically inspired problem-solving to enactive robotics

Enactivism was born under the conviction that robotics, as a field, would require, or even force, cognitive science to move beyond the problem-solving framework:

The assumption in CS [Cognitive Science] has all along been that the world can be divided into regions of discrete elements and tasks to which the cognitive system addresses itself, acting within a given “domain” of problems: vision, language, movement. Although it is relatively easy to define all possible states in the “domain” of the game of chess, it has proven less productive to carry this approach over into, say, the “domain” of mobile robots. Of course, here too one can single out discrete items (such as steel frames, wheels and windows in a car assembly). But it is also clear that while the chess world ends neatly at some point, the world of movement amongst objects does not. It requires our continuous use of common sense to configure our world of objects. (Varela, 1992, p.251)

Yet, moving away from the problem-solving paradigm has taken a long path, most of which, rather than abandoning problem-solving has deeply transformed the way we understand how nature solves those problems. In a sense, biologically inspired robotics has mostly followed the problem-solving approach and biological inspiration has focused on picking up biological mechanisms to solve problems: from the internal neuronal inspiration of artificial neural networks since its early conception (Rosenblatt, 1958) to their later development (Rumelhart et al., 1988) to the embodied strategies that either transform the problems to be solved by their “brains” or have outsourced the computational load of the problem solving to body and world (Pfeifer and Scheier, 2001). What radically distinguished biologically inspired robotics from GOFAI (Good Old Fashioned Artificial Intelligence) was a change of focus from the abstract to the concrete, from the symbolic to the sensorimotor and from the rational to the practical know-how of situated action. Despite the emphasis on self-organization, agent-environment emergence of behavioral functioning (Steels, 1990) embodiment and situated action (Maes, 1990), etc. the main goal was still to build robots capable of solving behavioral problems. After all, to put it with biologically-inspired roboticist Barbara Webb: “The sensorimotor problems faced by animals and by robots have much in common” (Webb, 1995, p. 117) and, not only can animals help us devise robots that solve problems in a biologically inspired manner, but also solving a sensorimotor problem with the robot could help us understand how the animal solves it; like “[d]etecting which ear is closer to the sound” which “is a non-trivial problem for the cricket” (Webb, 1995, p. 120).

Other trends of biological inspiration have built upon evolutionary theory itself and artificially evolved brains or brains and bodies to solve the problems (encoded as fitness function) in what is commonly known as evolutionary robotics (Cliff et al., 1993; Nolfi et al., 2016). Random variations to the parameters of robotic brain's and bodies are selected against a fitness function that operates as the benchmark of the problem to be solved. Despite the problem-solving focus that is almost inherent in artificial evolutionary optimization techniques, evolutionary robotics served to disclose a number of principles of behavioral self-organization that non-linear, fine grained agent-environment coupling display when artificial evolution can freely explore the solution space without the prejudices inherent to the human design (Harvey et al., 1997).

Some of these approaches entail radical departures from core assumptions of the computational functionalist theory of the mind: cognitive processing does not only occur in the head and the body must be integrated as a key feature of cognitive problem solving (not simply as an executioner of the solution representation worked out in the head or a sensory transmitter of the problem into it); agent-environment interactions can self-organize with little if any representations; material bodily and interactive constitution are not mere implementation details of abstract capacities but intrinsic part of the problems and solutions that cut across them.

But *enactive robotics* moves yet further into biological inspiration. On the one hand there are enactive approaches that have attempted to introduce more of the living body of natural intelligence into robotics by including self-organized mechanical, soft bodies (Man and Damasio, 2019) or even chemical bodies (Damiano and Stano, 2021). But also, and perhaps most relevant for enactive theory transferring to the robot what metabolism has to offer to anchor intrinsic needs or emotional feedback (Ziemke and Lowe, 2009). There is however another enactive path that brings into robotics some principles of living organization and, more specifically, the autonomy of behavior, a *way of life* for robots, not as somehow transferred from the biological body, but enacted at the scale of brain-body-world dynamics (Barandiaran and Moreno, 2006). Some forerunners of this inspiration are no doubt Ross Ashby on the organism-centered inspiration of adaptive controllers as machines capable to remain homeostatic in the face of perturbations (Ashby, 1952) and Grey Walter's “life imitating” robots “designed to illustrate the uncertainty, randomness, free will or independence so strikingly absent in most well-designed machines” (Grey Walter, 1950, p. 44).

More recent development of this line of inspiration on natural and biological principles for the design of robots came hand in hand with the development of a theory of autonomous behavior and agency (Smithers, 1997), organismically inspired robotics (Di Paolo, 2003), and habit-centered enactive robotics (Egbert and Barandiaran, 2014).

### 2.3. Enactivism and the autonomy of sensorimotor life

Varela, Thompson and Rosch opened up a new way of thinking in 1991. Their enactive approach conceives that “cognitive structures emerge from the recurrent sensorimotor patterns that enable action to be perceptually guided” (Varela et al., 1991, p.173). They later stated that: “[C]ognition is no longer seen as problem solving on the basis of representations; instead, cognition in its most encompassing sense consists in the enactment or a bringing forth of a world by a viable history of structural coupling” (Varela et al., 1991, p.205). The enactive approach thus emphasizes sensorimotor coupling and the recurrent patterns that emerge from agent-environment interactions.

This way Varela overcame the operational (en)closure of the nervous system that served as his main analogy with the organization of the living, captured (together with Humberto Maturana) within the theory of autopoiesis. Ever since, the relationship between the self-organizing nature of nervous activity and that of behavior became to some extent problematic (see Barandiaran, 2017 for a discussion).

Inspired by Maturana and Varela, Tim Smithers re-states the need for biologically inspired autonomy in robotics, in the context of the impossibility to design robot agents from a problem-solving stand point:

Designing and building autonomous agents thus becomes the problem of designing and building processes that can support and maintain this kind of identity formation through interaction: processes that, through interaction, are continuously forming the laws of interaction that can sustain and maintain the interaction needed to form them. In other words, we need interaction processes that can support the self-construction and maintenance of interaction processes through interaction, in essentially the same way that the material and energy interaction processes of single cells can be understood as being involved in the continual forming of the mechanisms that support this interaction. Such systems will thus be self-law making as well as self-regulating, in essentially the same way as we can understand biological systems and autonomous city states. (Smithers, 1997, p.102)

This analogy between metabolic autonomy and the autonomy of behavior was further explored in Di Paolo (2003). According to this view, enactivism needs not be committed to build bio-chemically living robots (provided that this is possible or even desirable) but to endow a robot with a *way of life*. This intuition was further explored in Barandiaran (2007, 2008).

Sensorimotor Contingency Theory can further enrich this approach. It uses regularities in the ways that motor activity affect sensory activity (sensorimotor contingencies) to explain the qualities of perceptual experience (O'Regan and Noë, 2001). Empirical research involving sensory substitution, sensory modification, psychophysics has informed the development of sensorimotor contingency theory (SMCT), which attempts to explain diverse aspects of perceptual experience, including why certain sensory modalities have a particular “feel” to them; how it is possible to make one experience one sensory modality (e.g., touch) in a way that feels more like another (e.g., sight); and the conditions in which subjects are (or are not) capable of adapting to major transformations to their sensorium. The key idea in SMCT is the role that action plays in perception: a classical enactive theme (Noë, 2004).

Using variations of the basic theme of how motor activity modifies sensory input, a set of robotic architectures where made using sensorimotor contingencies as building blocks for robotic design (Maye and Engel, 2013; Jacquey et al., 2019). But these are hardly enactive in the sense of the deep biological inspiration that the enactive approach can offer.

Perhaps the best way to explore such potential is to bring forth the concept of sensorimotor autonomy (updated and refined from a previous proposal of Mental Life and also explored in more detail on the concept of Sensorimotor Life): the capacity of an agent to sustain and regulate the structures that generate behavior. This definition echoes the metabolism-based definitions of life as far-from-thermodynamic equilibrium chemical systems capable of maintaining the network of chemical reactions that constitute it (Gánti, 1975; Maturana and Varela, 1980; Rosen, 1991; Ruiz-Mirazo et al., 2004; Luisi, 2006).

The basic constituent of sensorimotor autonomy is a sensorimotor structure (a behavioral scheme or habit) made possible by both a set embodied-neural pathways and a set of sensorimotor contingency relationships. Think of it as a coordination pattern that emerges out of environmental, sensorimotor and neural (or behavior generating) mechanisms. Now, if this structure is far-from-equilibrium or, said differently, if let alone it tends to extinguish or vanish, and if the very enactment of the sensorimotor scheme reinforces itself by repetition or by satisfying certain conditions that feed-back into its supporting structure (e.g., reinforcement of synaptic connections by Hebbian learning or reward reinforcement), then we have first sense of self-maintenance that is characteristic of habits. The more the habit is enacted the more it is strenghthened, the stronger it is the more likely it is to be enacted. That is the virtuous (or vicious!) self-sustaining nature of the habit.

We can now go back to the original, albeit obscure and self-referential, intuition of roboticist Tim Smithers and his idea of the autonomy of behavior based on the “processes that, through interaction, are continuously forming the laws of interaction that can sustain and maintain the interaction needed to form them.” These laws or norms are nothing other than the very conditions under which the habit is viable, that it can persist and sustain itself. This is a strong analogy with (metabolic) life that opens up the very possibility of *having a problem of your own* and *having to solve it*. The problem for the precarious, self-maintaining autonomous cell is persistence, avoiding decay and disintegration (see Barandiaran and Egbert, 2013 for a more detailed analysis). The same goes for the precarious, self-maintaining nature of a habit, and ultimately of autonomous sensorimotor life (see Barandiaran, 2008; Di Paolo et al., 2017). This approach also opens up a new mode of explanation that is characteristic of biological thinking and can be applied to sensorimotor dynamics: focusing on the nature and structure of constraints rather than the problems they are suppose to be adapted to solve. We return to these themes in the discussion section.

In what follows we introduce habit-based enactive robotics to illustrate and further develop the points we have briefly outlined. Inspired by SMC and enactive principles we build robots that are capable of generating spontaneously a complete ecology of habits that display structural constraints within the sensorimotor space. The results will help us discuss how enactive robotics can contribute to a new understanding of mind and cognition with a deeper biological inspiration than what problem-solving can provide.

## 3. Model

### 3.1. Overview

The computational model simulates a two-wheeled robot that moves around a two-dimensional environment. The robot has two independently controlled motors; one for each of its wheels, allowing it to move forwards or backwards in a straight line or to turn in a variety of arcs, or on the spot. The robot's motors are controlled by an iterant deformable sensorimotor medium (IDSM) (Egbert and Barandiaran, 2014)—a habit-forming controller that is described in detail below.

The robot's environment is periodic in the sense that when the robot moves off one side of the environment, it appears on the opposite side. A stimulus source is also located in the environment. This source, which moves around the environment, can be thought of as simultaneously emitting a sound tone and a source of light, but in any given trial the robot is only capable of perceiving one of those sensory modalities (light or sound). We now present each element of the simulation in detail.

The robot and its environment are simulated using Euler forward integration with a time step of Δ*t* = 0.01. Thus, a single time-unit consists of 100 iterations, and the IDSM is updated every iteration.

### 3.2. Stimulus

The stimulus source moves around the environment in a circle. Each rotation, it slows to a stop at its left-most position, before accelerating again to complete another rotation. This trajectory is specified by the following equations which describe the stimulus's position (*s*_*x*_, *s*_*y*_) as a function of time (*t*):

(1) $$\begin{array}{l} s_{x}=\frac{1}{2}+\cos\left(\frac{t}{10}+\sin\left(\frac{t}{10}\right)\right) \end{array}$$

(2) $$\begin{array}{l} s_{y}=\frac{1}{2}+\sin\left(\frac{t}{10}+\sin\left(\frac{t}{10}\right)\right). \end{array}$$

### 3.3. Robot

**Figure 1:**
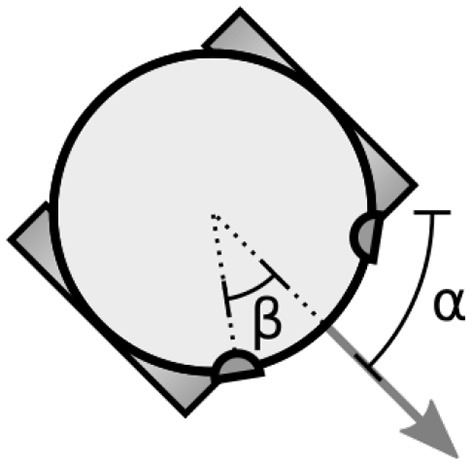
The simulated two-wheeled robot. The variable α specifies the orientation of the robot (direction of forward travel). The robot's sensors are located on its periphery with the parameter β specifying the offset of the sensors from the direction the robot is facing.

The simulated robot (Figure 1) has two independently controlled motorized wheels. It's position changes according to the following differential equations,

(3) $$\begin{array}{l} \frac{\text{d}x}{\text{d}t}=k_{s}\cos\left(\alpha \right)\left(m_{l}+m_{r}\right) \end{array}$$

(4) $$\begin{array}{l} \frac{\text{d}y}{\text{d}t}=k_{s}\sin\left(\alpha \right)\left(m_{l}+m_{r}\right) \end{array}$$

(5) $$\begin{array}{l} \frac{\text{d}\alpha }{\text{d}t}=k_{s}\frac{\left(m_{r}-m_{l}\right)}{2R}, \end{array}$$

where *x* and *y* are the robot's position in the environment; α is its heading; *k*_*s*_ = 0.25 scales the speed of the motors; *R* = 0.05 is the robot's radius; and the variables *m*_*l*_ and *m*_*r*_ represent the velocities of the robot's left and right wheel motors.

We consider two robot sensor configurations. “Visual” robots have two directional sensors. The excitation of each, *V*, is the product of an attenuation factor due to distance from that sensor to the stimulus, and an attenuation factor due to misalignment between the orientation of the sensor and the relative direction of the stimulus. This second attenuation factor is calculated by taking the scalar product of a unit vector that points from the sensor to the stimulus and $\vec{o}$, a unit vector that specifies the direction that the sensor is facing.

Formally,

(6) $$\begin{array}{l} V=\underset{\text{distance}}{\underset{︸}{\frac{0.25}{0.25+∥\vec{r}{∥}^{2}}}}\underset{\text{orientation}}{\underset{︸}{{\left(\frac{\vec{r}}{∥\vec{r}∥}\cdot \vec{o}\right)}^{+}}}, \end{array}$$

where $\vec{r}$ is a vector that describes the position of the stimulus relative to the sensor; ∥*r*∥ is the magnitude of that vector; and the + superscript indicates that negative values within the parentheses are truncated to zero. The excitation of these sensors is thus highest when the sensor is close to the stimulus and directly facing it.

“Auditory” robots have two sensors that respond to the rate at which the sensor is approaching or moving away from the stimulus source. This is analogous to the Doppler effect whereby the perceived frequency of a sound when approaching is higher than when moving away from the listener. The excitation of an auditory sensor, *A*, is given by:

(7) $$\begin{array}{l} A=\frac{1}{2}+k\frac{\text{d}||r||}{\text{d}t} \end{array}$$

where the first term can be thought of as the tones natural pitch which is offset by the relative speed of the sensor and the stimulus scaled by $k=\frac{3}{4}$ to keep the magnitude of the sensor (given the relative speeds of the robot and the stimulus) within a similar range of excitation values as simulated for the visual sensor.

To address the periodic boundaries of the environment, auditory sensors always use the nearest stimulus source as defined by the minimum image convention and visual sensors calculate the combined effect of 5 stimuli: one in the simulated space and four virtual copies of the sensor offset north, south, west and east of the simulated space arena by one arena width. This means that if, for example, a visual robot is close to the north boundary of the arena and facing north it can still see stimuli.

Visual sensors are offset from the direction the robot is facing by β_*v*_ = π/5 (see Figure 1). Auditory sensors are offset by β_*a*_ = π/2. The orientation of visual sensors is α ± β_*v*_, i.e., perpendicular to the tangent of the robot's circular body at that position, facing outwards. Auditory sensors have no orientation.

### 3.4. IDSM

#### 3.4.1. Overview

The IDSM is a robot controller intended to capture the idea of a self-maintaining pattern of sensorimotor activity. Inspired by the habitual behavior of people and by the enactivist concept of “*autonomous”* self-sustaining sensorimotor systems (see e.g., Di Paolo et al., 2017), the IDSM was designed such that patterns of sensorimotor activity reinforce the mechanisms that produce them (Egbert and Barandiaran, 2014; Egbert and Cañamero, 2014; Egbert, 2018). The IDSM has been used to explore how a habit-based individual can be trained to perform different tasks (Egbert and Barandiaran, 2014); how different forms of motor babbling can bias the subsequent formation of habits (Zarco and Egbert, 2019); how the essential variables of a biological autonomous system can be shared with the essential variables of a sensorimotor autonomous system (Egbert and Cañamero, 2014) and the extent to which IDSM-based sensorimotor autonomous systems can be considered to be adaptive (Egbert, 2018). In the present paper, we use the IDSM in a new way: to show, in a formal model, how sensorimotor-contingencies can play an essential role of sculpting the form of habits without themselves being explicitly internalized or represented by the “brain” or “controller” of an embodied agent.

The IDSM works by recording trajectories taken through sensorimotor space, i.e., how the sensorimotor state changes for various experienced sensorimotor states. When a sensorimotor state is experienced that is similar to one that has been experienced in the past, the motors of the robot are actuated in a way similar to how they were actuated in that previous experience. Memories of previous trajectories are gradually forgotten, unless they are reinforced by re-enactment and so the only patterns of behavior that can persist for long periods of time are those that are re-enacted. When the sensorimotor state is in an unfamiliar (or forgotten) state, motor activity is random. The IDSM used in this paper is very similar to that described in Egbert (2018). Any differences between the model here and that in Egbert (2018) are explicitly highlighted below.

A useful metaphor for understanding how the IDSM works is the paths that form on university campuses, where paths taken by students crossing a grassy field between academic buildings trample and kill the grass. The emergent dirt paths influence the trajectories taken by subsequent students, but the grass also regrows, so only emergent paths that are regularly traveled can persist in the long-term. This is essentially how the IDSM operates, but the trajectories taken and the paths that form are in sensorimotor space, rather than on a university campus. The dynamic also relates to the self-reinforcing nature of habitual behavior, where repeated performance of patterns of behavior (e.g., the direction you look when crossing the street; smoking a cigarette; or a tendency to worry) increases the likelihood of similar behavior being performed in the future. And to reiterate: the IDSM was designed to capture the enactivist concept of autonomy (a precarious self-maintaining system) in a sensorimotor system—see Di Paolo et al. (2017).

More formally, the IDSM can be thought of as a function, *f*, that transforms the robot's current sensorimotor state into an “output,” i.e., the next moment's motor state: *f*_*t*_(*S*_*t*_, *M*_*t*_) → *M*_*t*+1_. As this function is applied, the function itself also changes as a function of the current state of sensors and motors and the current state of the function: $\frac{\Delta \text{f}}{\Delta \text{t}}=g\left(f,S,M,\frac{\Delta S}{\Delta \text{t}},\frac{\Delta M}{\Delta \text{t}}\right)$. This change, which we shall now describe, was engineered so that sensorimotor state trajectories would bias the system to increase the likelihood that similar sensorimotor trajectories will be repeated in the future.

#### 3.4.2. Tracking sensorimotor trajectories

As the robot's sensorimotor state changes, the IDSM maintains a set of records called “*nodes.”* Each node describes the sensorimotor-velocity (i.e., the rate of change in all sensors and motors) for a particular sensorimotor-state at the moment that the node was created. Each node, *N*, is a tuple, *N* = 〈***p***, ***v***, *w*〉, where ***p*** represents the sensorimotor state associated with the node (referred to as the node's ‘position in sensorimotor space’); ***v*** indicates the sensorimotor velocity when the node was created; and *w* indicates the weight of the node, a value that changes dynamically and is used to scale the overall influence of the node. We shall refer to these components using a subscript notation, where the position, SM-velocity vector, and weight of node *N* are written as *N*_***p***_ and *N*_***v***_ and *N*_*w*_, respectively.

As a robot controlled by the IDSM moves through sensorimotor states, new nodes are created recording the sensorimotor velocities experienced at different sensorimotor states. Specifically, when a new node is created, its *N*_***p***_ is set to the current sensorimotor state; its *N*_***v***_ is set to the current rate of change in each sensorimotor dimension, and its initial weight, $N_{w}^{0}$.

**Figure 2:**
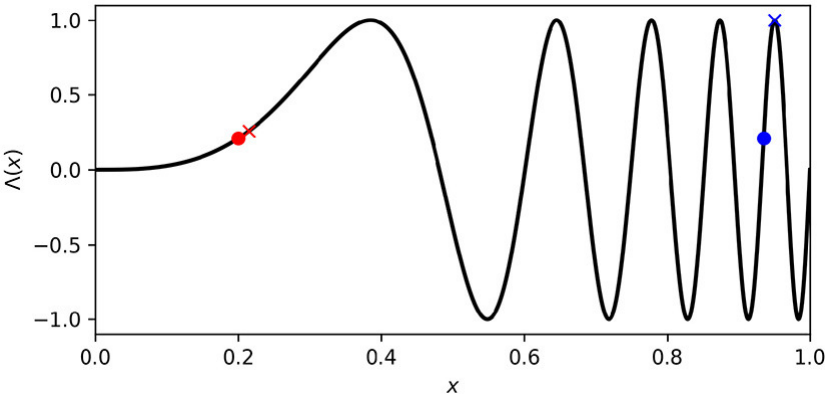
Abstract motor-state to motor-state mapping. Changes made to low abstract motor state [e.g., red circle and (X)] produce less change in actual motor state (Λ(*x*)) than when the abstract motor state is high (e.g., blue circle and X).

The two vector terms (*N*_***p***_ and *N*_***v***_) can be thought of as existing within an abstract sensorimotor space (ASMS). A many to one mapping transforms any given position in the ASMS to a specific sensorimotor state, according to:

(8) $$\begin{array}{l} \left[\begin{array}{c} \Lambda \left(\mu_{l}\right)\\ \Lambda \left(\mu_{r}\right)\\ \sigma_{l}\\ \sigma_{r} \end{array}\right]=\left[\begin{array}{c} m_{l}\\ m_{r}\\ s_{l}\\ s_{r} \end{array}\right] \end{array}$$

where μ_*l*_, μ_*r*_, σ_*l*_, and σ_*r*_ indicate a position in the four dimensional ASMS; the vector on the right indicates the sensorimotor state (i.e., the actual state of left-motor, right-motor, left-sensor, and right-sensor) associated with that ASMS position; and Λ(*x*) = sin[(4+*x*)2π*x*^3^] is the non-linear function plotted in Figure 2. The purpose of this mapping is to avoid prescribing a characteristic rate of motor change, and instead to allow the IDSM to autonomously find habits with rates of motor change that are neither too fast nor too slow. Different regions of ASMS correspond to different rates of motor change. For instance, when abstract motor state *x* ⪅ 0.5, a small change in that state variable corresponds to a small change in Λ(*x*), the actual motor state (see the difference between the red circle and red X in Figure 2), where an equivalent change in *x* when *x* ⪆ 0.7 corresponds to a greater change in the actual motor state (blue circle and X in Figure 2). When the sensorimotor state of the robot is unfamiliar, changes in motor activity are driven randomly (as explained below) and this Λ mapping allows these random changes in sensorimotor space to correspond to slow or fast changes in motor state. By exploring different parts of the abstract sensorimotor state, the IDSM can experiment with sensorimotor patterns with different rates of change until ones that are self-reinforcing emerge. The ASMS is also treated as periodic so as to avoid the IDSM getting stuck at motor boundaries (as discussed in Egbert, 2018). ASMS state variables, μ_*l*_, μ_*r*_, σ_*l*_, σ_*r*_ all ∈ [0, 1] and the ASMS distance functions (described below) adhere to the minimum-image convention.

New nodes are added when the density of nodes near the current sensorimotor state is less than a threshold value, i.e., when ψ(***x***) < *k*_*t*_. Loosely speaking, ψ is a measure of how ‘familiar’ the current sensorimotor state is, as estimated by summing a non-linear function of the distance (Equation 11) from every node with a positive weight to the current sensorimotor state. Formally,

(9) $$\begin{array}{l} \psi \left(x\right)=\underset{N}{\sum }\omega \left(N_{w}\right)d\left(N_{p},x\right) \end{array}$$

(10) $$\begin{array}{l} \omega \left(N_{w}\right)=\{\begin{array}{ll} 1 & \text{if }N_{w}>0\\ 0 & \text{otherwise} \end{array} \end{array}$$

where ***x*** represents the current ASMS position, *k*_*t*_ is a threshold parameter describing maximum node-density at which new nodes will be created and *d*() is the following non-linear ASMS-distance function.


(11) $$\begin{array}{ll} d\left(N_{p},x\right)= & \frac{2}{1+\text{exp}\left(k_{d}||N_{p}-x|{|}^{2}\right)} \end{array}$$

           
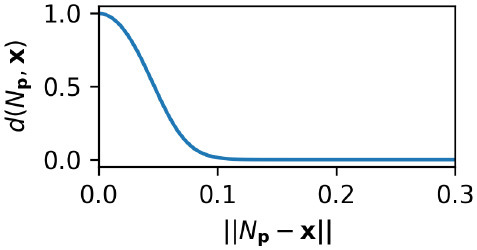


#### 3.4.3. Nodes influence the sensorimotor state

One time unit after a node has been created, it is added to the set of ‘activated’ nodes that influence the sensorimotor state according to:

(12) $$\begin{array}{l} m=\frac{1}{\phi \left(x\right)}\underset{N}{\sum }\omega \left(N_{w}\right)d\left(N_{p},x\right){\left(\underset{\text{vel.}}{\underset{︸}{N_{v}}}+\underset{\text{attraction}}{\underset{︸}{A\left(N_{p}-x,N_{v}\right)}}\right)}^{\mu } \end{array}$$

This equation describes a weighted average of the influence of all of the nodes. The influence of each individual node is the sum of its “velocity” factor and its “attraction” factor. The velocity factor is simply the *N*_***v***_ vector (i.e., the sensorimotor velocity recorded when the node was created). The attraction factor is defined by

(13) $$\begin{array}{l} A\left(a,N_{v}\right)=a-\left(a\cdot \frac{N_{v}}{\left||N_{v}|\right|}\right)\frac{N_{v}}{\left||N_{v}|\right|} \end{array}$$

and it causes the sensorimotor state to move toward the node. The attraction term is included to cause the system to move toward more familiar regions of sensorimotor space and to help stabilize patterns of repeated behavior (see Egbert and Barandiaran, 2014, 2015). The μ superscript in Equation (12) expresses that the IDSM only (directly) controls the motor components of the sensorimotor state. The sensory components are the result of the robot's relation to its environment and so are not directly controlled by the IDSM, but are, of course, *influenced* by the motor dynamics, indirectly through the sensorimotor contingencies determined by the robot's environment and body.

The influence of each node is attenuated by a non-linear function of the distance between the node and the current sensorimotor state. This attenuation is expressed by the term *d*(*N*_***p***_, ***x***) and it means that nearby nodes affect the sensorimotor state and farther away nodes have little influence. The influence is also attenuated by a threshold function of weight, [ω(*N*_*w*_)] such that only positively weighted nodes affect the motor state. Previous versions of the IDSM had a more complicated sigmoidal function in place of the simpler threshold function used here (Equation 10). Note that the degradation of the nodes and the threshold function of Equation (10) mean that when nodes are not visited for a long period of time they cease to have any influence whatsoever. Nodes that have degraded to this point essentially cease to exist.

After a node is created, its weight changes according to:

(14) $$\begin{array}{l} \frac{\text{d}N_{w}}{\text{d}t}=-k_{↓}+k_{↑}d\left(N_{p},x\right) \end{array}$$

In this equation, the first term represents a steady degradation of the node's influence and the second term represents a strengthening of the node that occurs when the current sensorimotor state is close to the node's position.

The influence of all nodes is summed and then scaled by the local density of nodes,

(15) $$\begin{array}{l} \phi \left(x\right)=\underset{N}{\sum }\omega \left(N_{w}\right)d\left(N_{p},x\right){|}_{\text{activated nodes}} \end{array}$$

This equation looks similar to that used to calculate ψ (Equation 9), but is different in that ψ describes the local density of all nodes, where as ϕ describes the local density of activated nodes *only*.

#### 3.4.4. Random motor activity

When the local density of activated nodes is low, motor behavior is random. This is accomplished by defining of a “switch” value, *s*, which specifies when the behavior is to be random and when it is to be controlled by the influence of the IDSM's nodes. The following equation expresses that *s* is 1 when ϕ is low and 0 when ϕ is high; and that it moves between these values in a smooth, sigmoidal manner:


(16) $$\begin{array}{ll} s= & \frac{1}{1+\text{exp}\left(R_{g}\left(\phi \left(x\right)-R_{t}\right)\right)} \end{array}$$

           
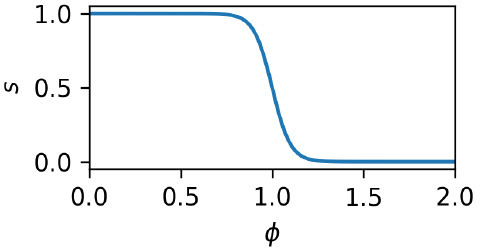


Here, *R*_*g*_ and *R*_*t*_ are parameters that specify the threshold of familiarity and the discreteness of the transition between random and non-random motor activity. This value is then used to switch between random motor activity (***r***), and IDSM's output (***m***), thus:

(17) $$\begin{array}{l} \frac{\text{d}\mu }{\text{d}t}=\left(1-s\right)m+sr. \end{array}$$

where the random motor activity vector, ***r*** is varied over time to produce a random walk in motor space as follows: every iteration, there is a *R*_*p*_ chance that the components of ***r*** will be assigned random values selected from a normal distribution with a standard deviation of *R*_σ_. The value of the *R*_σ_ and other parameters can be found in Table 1.

**Table 1:** Parameters.

**Parameter**	**Value**	**Description**
**ROBOT**		
*R*_*s*_	0.25	Robot speed
*R*_*d*_	0.1	Robot diameter
*R*_*o*_	π/5	Offset of sensors from center line of robot
*R*_*f*_	0.25	Falloff of sensor excitation with distance
**LIGHT**		
*L*_*v*_	0.1	Light velocity scale constant
*L*_*r*_	1/3	The radius of the light's circling motion
**IDSM**		
*k*_*d*_	500	Fall-off of non-linear ASMS distance measure
*k*_↓_	5	Node weight decay rate
*k*_↑_	2000	Node-reinforcement rate constant
*k*_*t*_	1	Node-density threshold parameter, influencing when new nodes are to be added
$N_{w}^{0}$	1000	Node weight upon creation
**MOTOR BABBLING**		
*R*_*p*_	0.1	Chance of random reassignment of random-motor walk direction and velocity
*R*_σ_	0.25	Standard deviation of random motor components
*R*_*t*_	1	Node-density threshold parameter, influencing when motor activity is random
*R*_*g*_	20	Steepness of transition between random and IDSM driven motor activity

## 4. Experiments and results

We now present two computational simulations of this model where we vary the sensory modality of the robot to explore how sensorimotor contingencies constrain the forms of the sensorimotor habits that can emerge and self-stabilize. The simulated robots and their environments are identical except that one robot's sensors are visual (as described above) and the other's are auditory. Formally, the only difference between these simulations is the equations that describe the stimulation of the robot's left and right sensors (Equations 6, 7).

To generate the data presented below, we ran 10 trials of each condition (i.e., we simulated 10 auditory robots and 10 visual robots) and for each condition, we selected a trial that displayed a wide variety of habits, and for which the simulated agent returned to one or more habits that it performed earlier but had stopped performing for some period of time. Not all trials did this—some instead rapidly fell into a pattern of behavior that was stable for the duration of the simulation. Data for all of the simulations is available at [DATA STORE LOCATION]. The analysis below covers visual simulation #9 and auditory simulation #0.

We now present an overview of the behaviors demonstrated by these two robots.

### 4.1. Visual sensors

**Figure 3:**
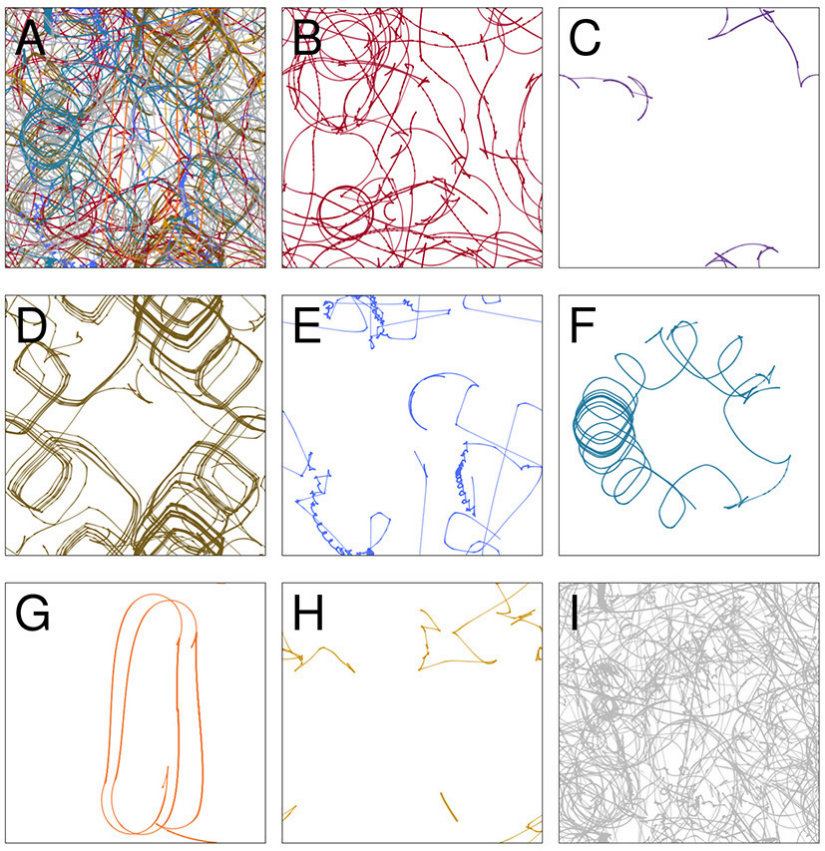
The trajectory taken by the visual robot **(A)**, broken down into 8 patterns of repeated sensorimotor activity, i.e., habits **(B–H)**. The portions of the trajectory where the robot's behavior is not clearly repetitious are included in plot **(I)**. Each of the nine plots shows the full 1 × 1 arena. Video showing this trial is available at https://www.youtube.com/watch?v=2v1TyvKz9qw.

**Figure 4:**
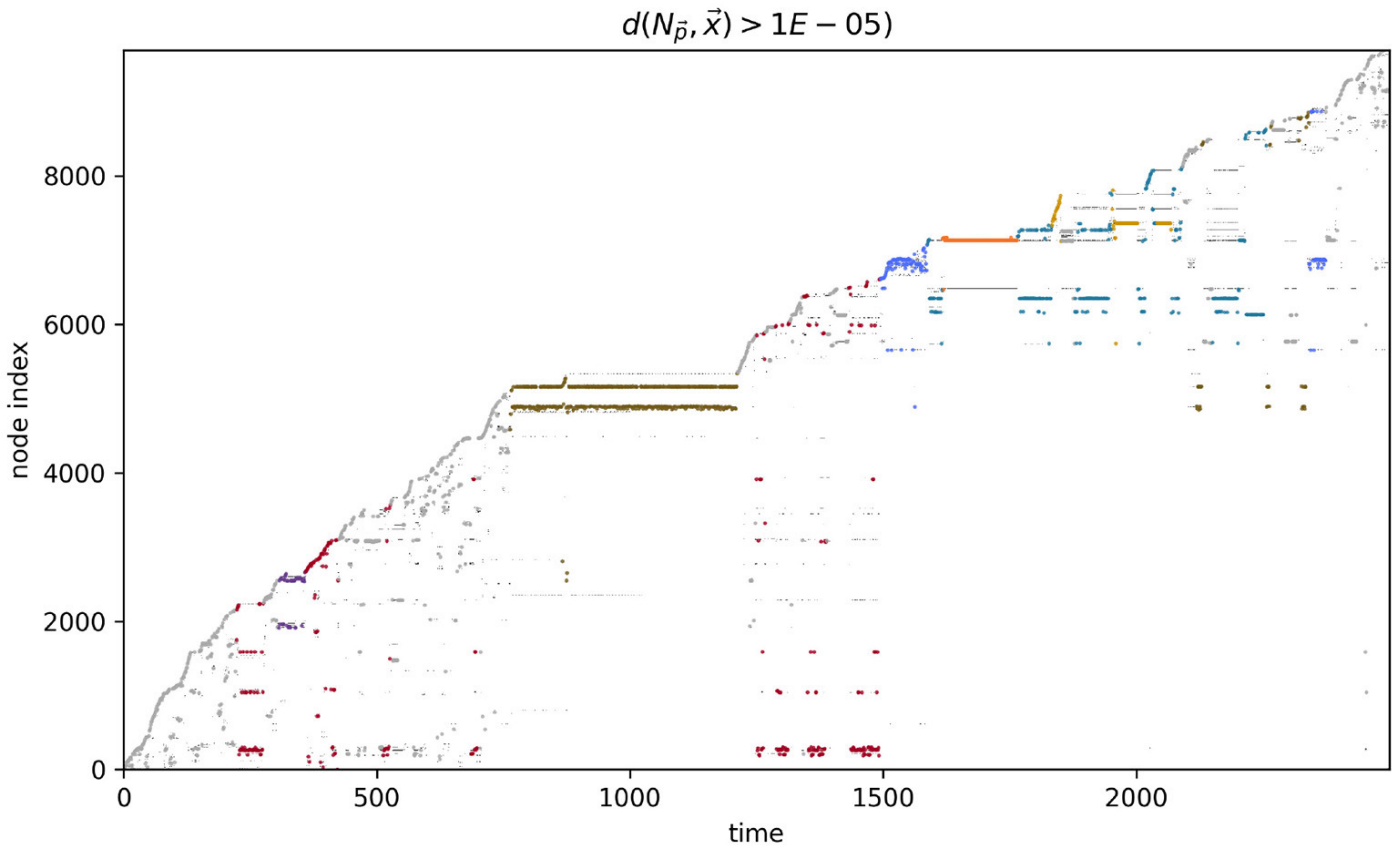
Node proximity during the course of the visual robot's simulation. The darker the point in this plot, the closer that node is to the robot's sensorimotor state at that point in time. The larger colored points indicate the index of the node that was closest (in sensorimotor state) to the robot's sensorimotor state at that time. The color of the closest-node points indicates the habit that those nodes are associated with. These colors match those in Figure 3.

**Figure 5:**
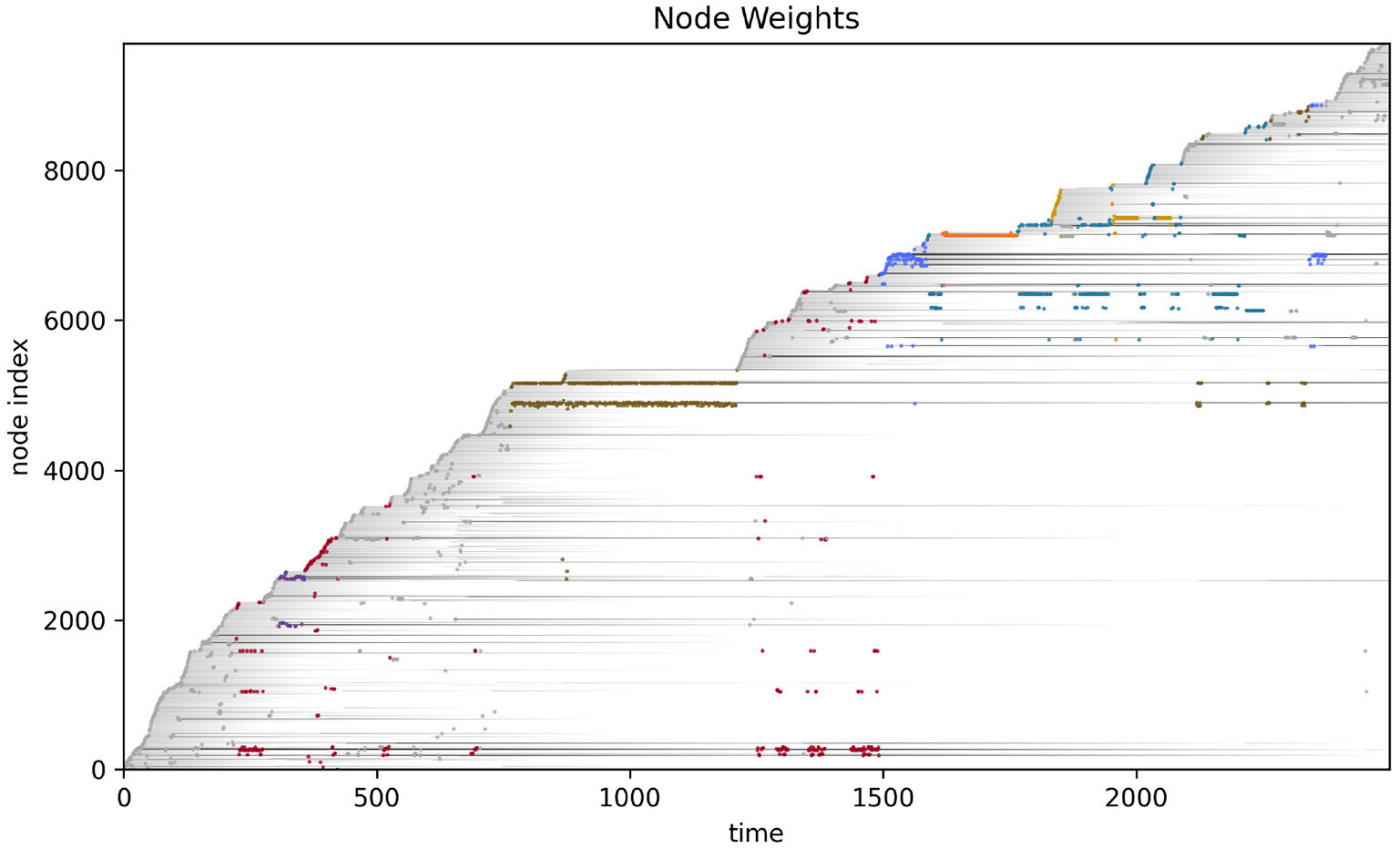
State of sensors and motors for the simulation of the visual robot.

The path taken by the visual robot through its environment is displayed in Figure 3A. This is a complicated and difficult to visualize trajectory, but it in fact involves several distinct repeated patterns of sensorimotor behavior (Figures 3B–H). To identify these, we first plotted the proximity of each of the IDSM's nodes to the robot's sensorimotor state over the course of the simulation (Figure 4). In this time series, it is easy to observe segments of time in which a particular set of nodes is repeatedly visited. For instance, when 750 ⪅ *t* ⪅ 1,200, there are a few nodes with indices close to 5,000 that are repeatedly visited (tan horizontal sequence of dots), and the same set of nodes are briefly revisited three times in the final 500 time units of the simulation. We manually identified these repeated patterns of sensorimotor activity, and assigned each pattern a label and a color (the example just provided is labeled “D” and colored tan). Times when the robots behavior is not clearly repeated were not assigned a label and are marked with a light gray color. To be clear, repetitions were identified in sensorimotor space (*via* the identification of repeatedly visited nodes), not in physical space. Returning to Figure 3, we can see that when the robot is performing the D pattern, it is moving around the environment, regularly turning in loops with squarish corners. Figure 5 shows how the state of the sensors, motors and the distance between the stimulus and the robot as the trial progresses. We can see that each of the colored regions tend to occupy particular regions of the sensorimotor space (the sensorimotor ‘habitat’ of the habit—see Buhrmann et al., 2013) and certain patterns of behavior involve the robot pursuing the moving light (e.g., F-behavior) while others avoid it (e.g., D-behavior).

Each of patterns B–H can be construed as a potential habit, i.e., a pattern of autonomous, self-maintaining sensorimotor activity. To justify this claim, we observe that

1. A node can persist for an extended time if and only if it is regularly “visited,” i.e., if and only if the robot's sensorimotor state regularly comes sufficiently close to the node such that the node's weight always kept above zero.
2. The robot's sensorimotor trajectory depends upon the nodes in that (a) the nodes directly control how the motor components of the sensorimotor state change, and (b) the nodes indirectly constrain and influence the sensory components *via* their effect upon the motors.

From these observations it follows that the nodes enable the very thing that they depend upon to persist and reflexively, the enactment of the pattern of behavior enables the very thing that it depends upon (the nodes) for its persistence. Similar to a model of biological autopoiesis (Varela et al., 1974), where inherently unstable components such as metabolism and membrane are mutually enabling, the components of these patterns of behavior in this model are in a relationship of mutual interdependence and support, and thus can be considered autonomous under some readings of the enactivist literature. The behavior itself is operationally closed entity (a “unity”), constituted by inherently unstable components (the nodes and the sensorimotor trajectory) and yet persists thanks to its own enactment or performance.

**Figure 6:**
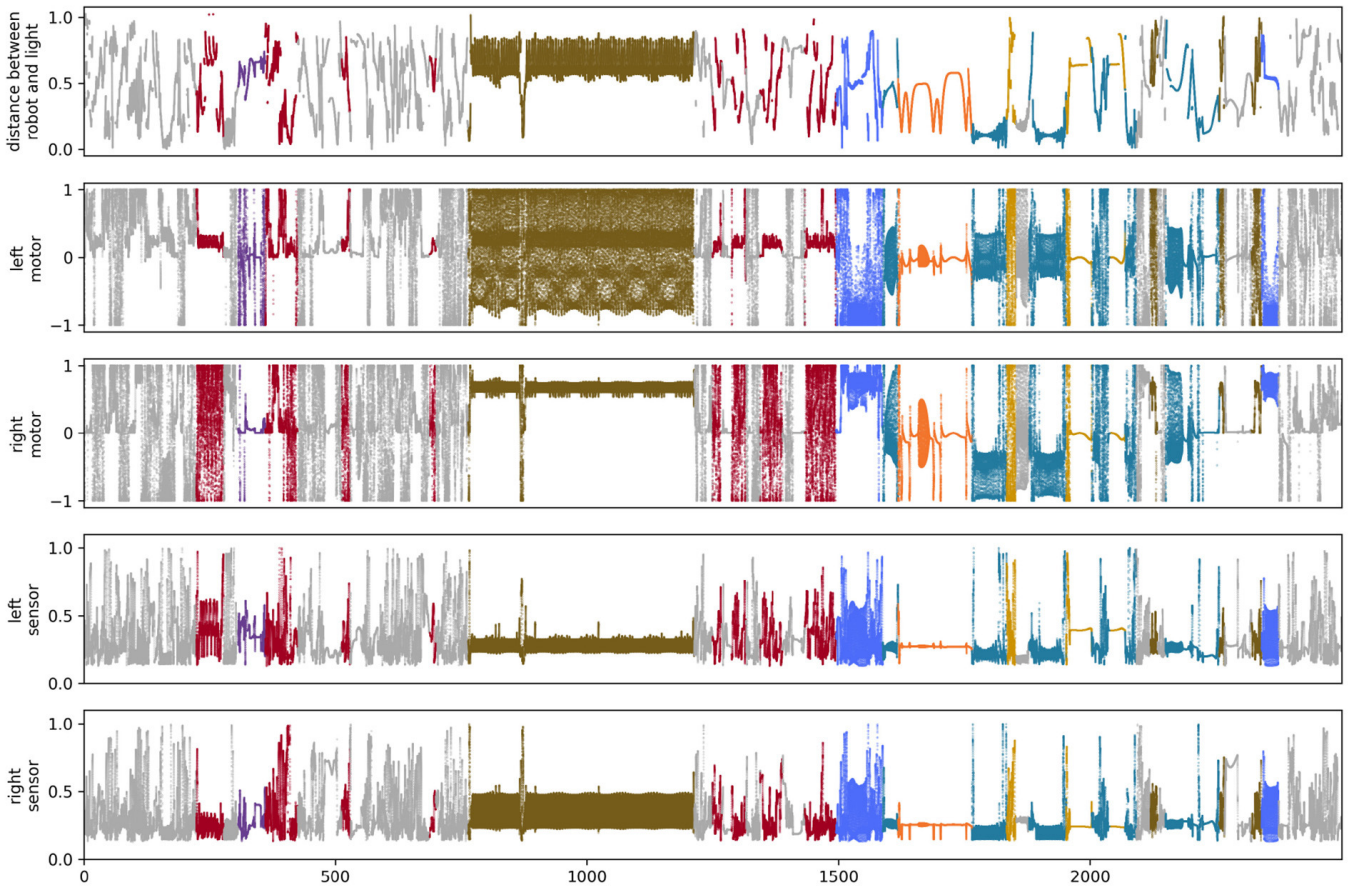
Node weight during the course of the visual robot's simulation. The weight of each node (shades of gray) are shown as time progresses during the simulation. After most nodes appear, their weights decay (become lighter as time passes), but some nodes are regularly visited and their weights are reinforced (long gray horizontal lines). The closest node at any time is plotted in color, just as in Figure 4.

More detailed analysis of the operational closure of these systems is outside the scope of the present paper (but see Egbert, 2018 for initial analysis of the precarious autonomy of a simple IDSM-based habit). We can see the basic idea however, when we consider how the weight of the nodes changes as time passes in the simulation (see Figure 6). Recalling that when the weight of a node reaches zero, that node ceases to exist, it is clear that only nodes that are regularly visited can persist in the long term. For a collection of nodes to be visited, the sensorimotor trajectory must move in a particular trajectory and the sensorimotor trajectory is largely determined by the activity of nodes. It follows from this that the only way that a pattern of behavior can persist is if it is one that causes the repeated revisitation of its constituent nodes. We can also note that this self-reinforcement of a habit need not be constant or contiguous. For example, the B-habit (red) is established early in the simulation [*t* ≈ 250)] and then is not visited again until *t* ≈ 1,250, where it is enacted a few times and the nodes are reinforced such that they survive until close to the end of the simulation. The parameters that prescribe the rates of node weight reinforcement (*k*_↑_) and degradation (*k*_↓_) determine how regularly patterns of behavior must be enacted if they are to persist.

### 4.2. Auditory sensors

**Figure 7:**
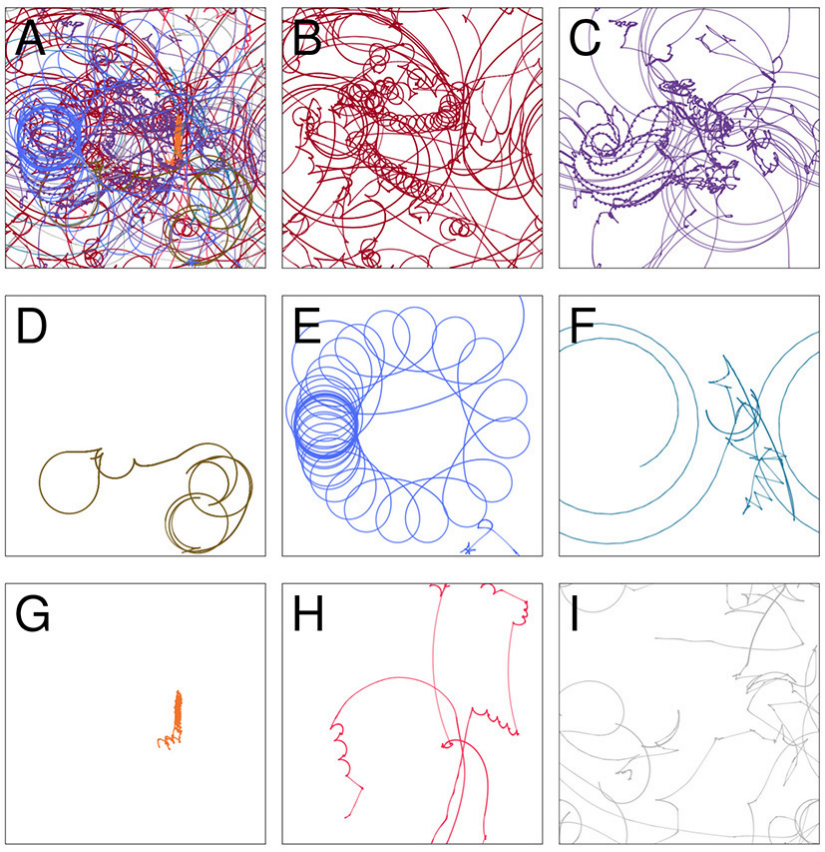
Spatial trajectory taken by the auditory robot **(A)**; broken down into “habits” i.e., self-reinforcing patterns of behavior **(B–H)**; and portions of the trajectory not associated with any habits **(I)**. Each of the nine plots shows the full 1 × 1 arena. Video showing this trial is available at https://www.youtube.com/watch?v=If_WeclEtCM.

**Figure 8:**
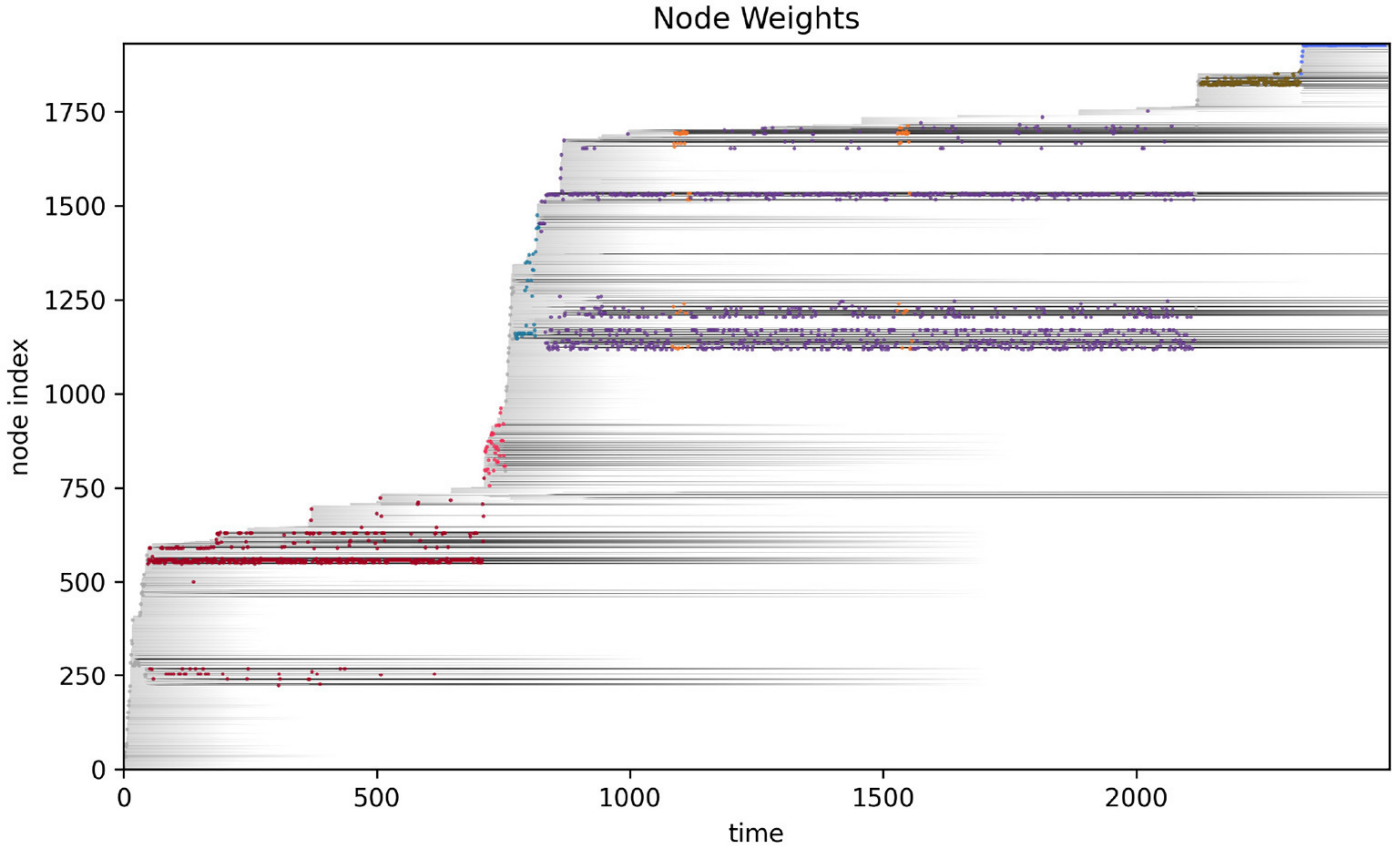
Node weights with nodes closest to the current sensorimotor state of the auditory robot highlighted and marked by color of habit.

**Figure 9:**
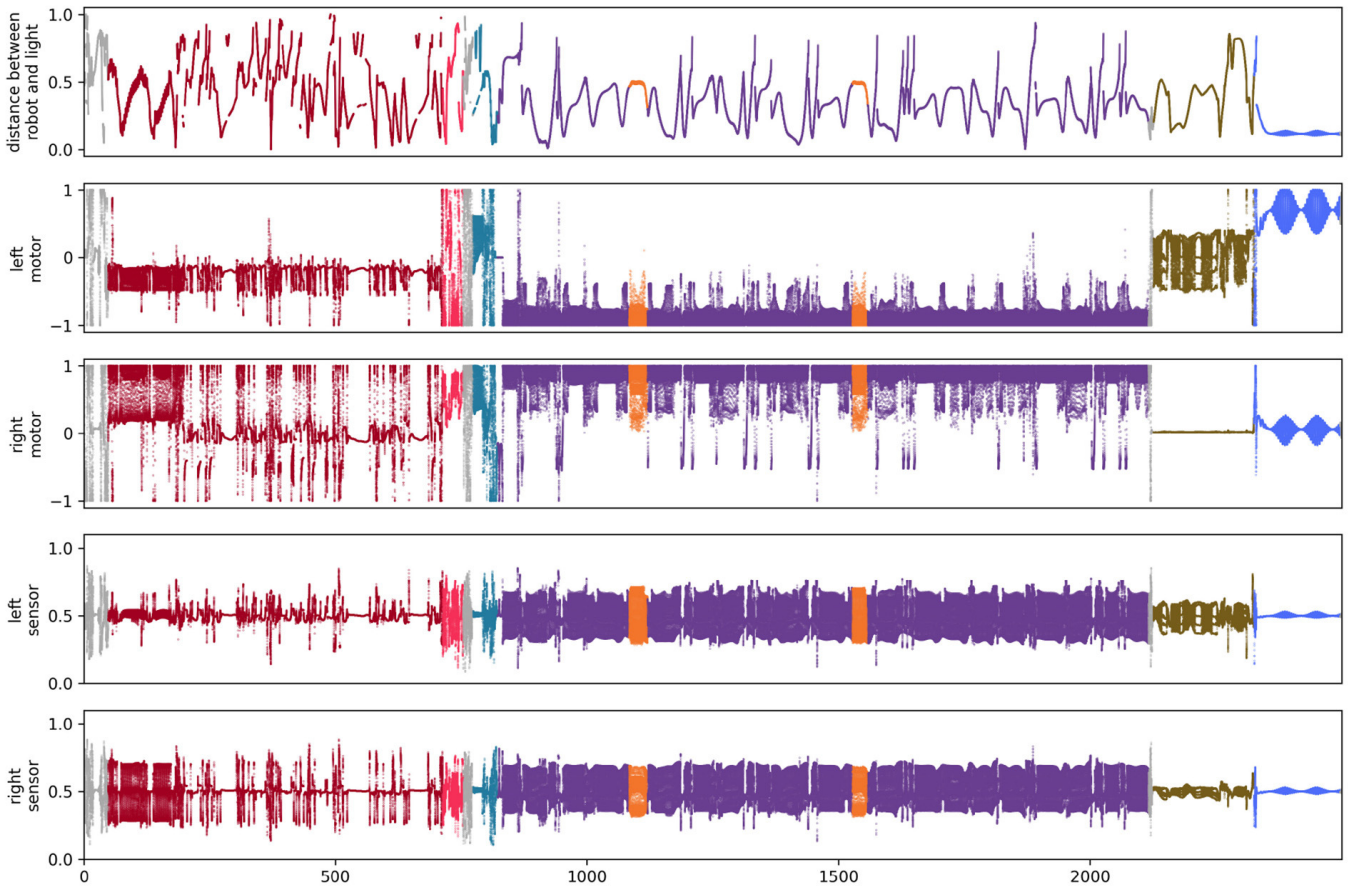
State of sensors and motors for the simulation of the auditory robot.

We performed a similar analysis on the auditory robot. Figure 7A shows the complete path taken by the auditory robot in its environment, broken down by habit (Figures 7B–H). Figure 8 shows the weight of each node which also provides an indication for how close each node is to the current sensorimotor state of the robot and the timeseries plots for the auditory robot are shown in Figure 9. It is worth noting that though these patterns of behavior may seem random, they all (both visual and auditory) relate to the moving stimulus source in regular, non-random ways. The regularities in the interaction with the stimulus are much more easily seen in the animations linked to in the captions of Figures 3, 7).

### 4.3. Comparison of sensorimotor structures

**Figure 10:**
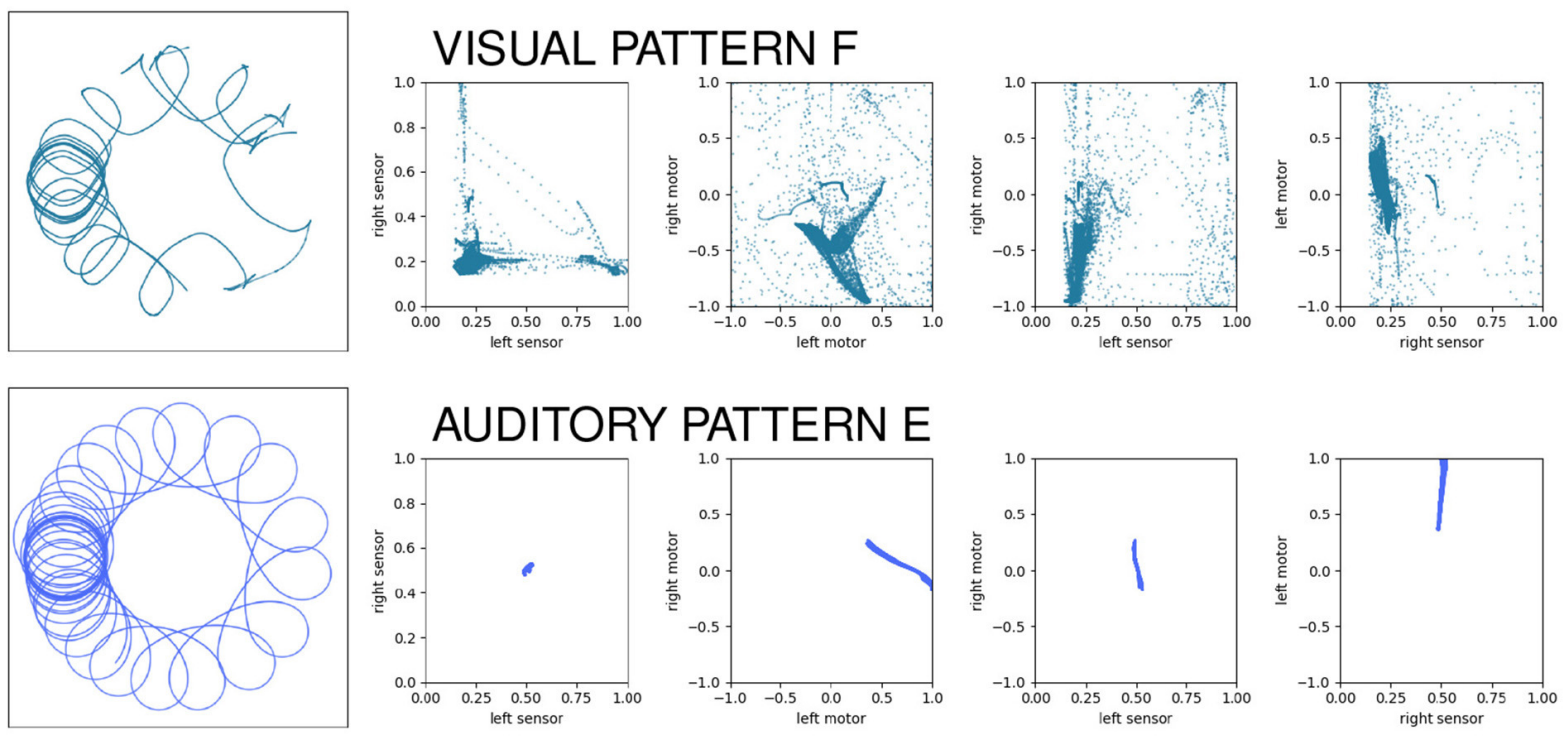
Contrasting the sensorimotor dynamics of two “circling” habits. In both cases, the robot circles the stimulus, but they use different sensory modalities to do so. The patterns of sensorimotor activity are clearly different despite the external “functional” description of the behavior.

Even when two behavior's functions are the same (e.g., they both accomplish taxis) the forms of the underlying sensorimotor habit can be different. As a case in point, we can compare auditory habit E with visual habit F. In both cases, the robot circles around the stimulus, maintaining a similar approximate distance from the stimulus as the stimulus moves in spurts around the environment. When we look at the two patterns of sensorimotor activity (Figure 10), we see two different pictures. Each row of this visualization shows the spatial trajectory of the robot (left) followed by four projections of the 4D sensorimotor trajectory of the robot and it is obvious that the two sensorimotor trajectories are qualitatively different. The auditory pattern involves less diversity in the range of states visited; the mean and other statistical properties of the sensorimotor trajectories are also clearly different, etc. But every habit in this model is unique—how much of the difference between these habits is simply due to the fact that they are different habits, and how much is due to the difference in the robot's sensorimotor modalities?

To address this question, we can look at projections of the sensorimotor trajectories of all of the habits of the visual robot (Figure 13) and observe that there are regularities in the forms of the habits of this robot. This is most readily seen in the left-sensor vs. right-sensor projection (upper right) which reveals elements of this robot's sensorimotor contingencies, including aspects of both its sensorimotor habitat and environment (Buhrmann et al., 2013) of the robot. Specifically, the bilateral symmetry of the robot's embodiment result in a (statistical) mirror symmetry across the left sensor = right sensor diagonal. The directionality of the visual sensors and their different orientations mean that it is impossible (in the environment with a single stimulus) to maximally stimulate both left and right sensors concurrently, leading to the L-shape apparent in upper-right plot of Figure 13, with arcs that pass between the stems of the L as the robot turns such that the stimulus moves from being in front of one sensor to in front of the other. These features are also found in the other nine visual robot trials.

Similar plots for the auditory robots (Figure 14) also reveal regularities. Like the visual robot, the auditory robot's embodiment is bilaterally symmetric and so it has the same symmetry across the diagonal in left-sensor/right-sensor plot, but the non-directionality of the auditory sensors and the fact that their excitation does not fall off with distance make other aspects of the sensorimotor activity different. The auditory trajectories tend to repeat patterns of diverging from a center point in short arcs in a pattern of returning and diverging again, in a different direction. These dynamics can be seen in greater detail in Appendix A, which shows the projection in sensorimotor space of each habit individually.

Sensors and motors are involved in a recursive relationship of influence, where sensors influence motors (*via* the controller) and motors influence sensors (*via* the effect of actions upon the environment and the agent's relationship to the environment). It therefore would make sense that the constraints imposed upon sensory dynamics by different sensory modalities would constrain the motor dynamics. In other words, under one sensory modality, certain motor trajectories will be more readily repeated (and thus stabilized) than others, and which motor trajectories are more readily repeated would depend upon the sensory modality.

**Figure 11:**
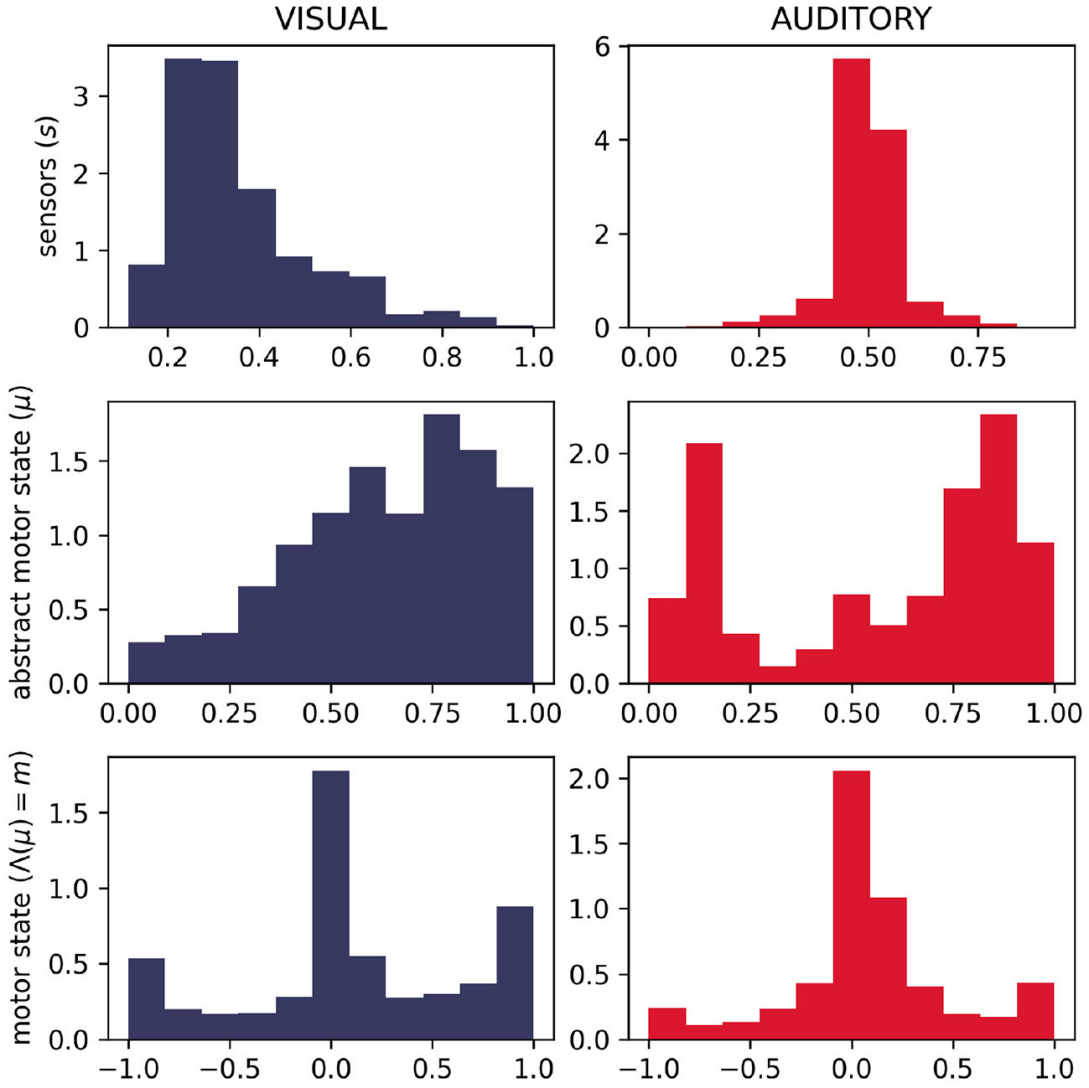
Histograms showing difference in distributions of sensorimotor states for 50 visual and 50 auditory trials. As expected, the different sensory modalities produce different distributions of the sensor state. It can also be seen that in visual trials, the abstract motor-state tended to be higher (corresponding to faster rates of motor state change—see Figure 2) and the motor state tended to be more extreme.

**Figure 12:**
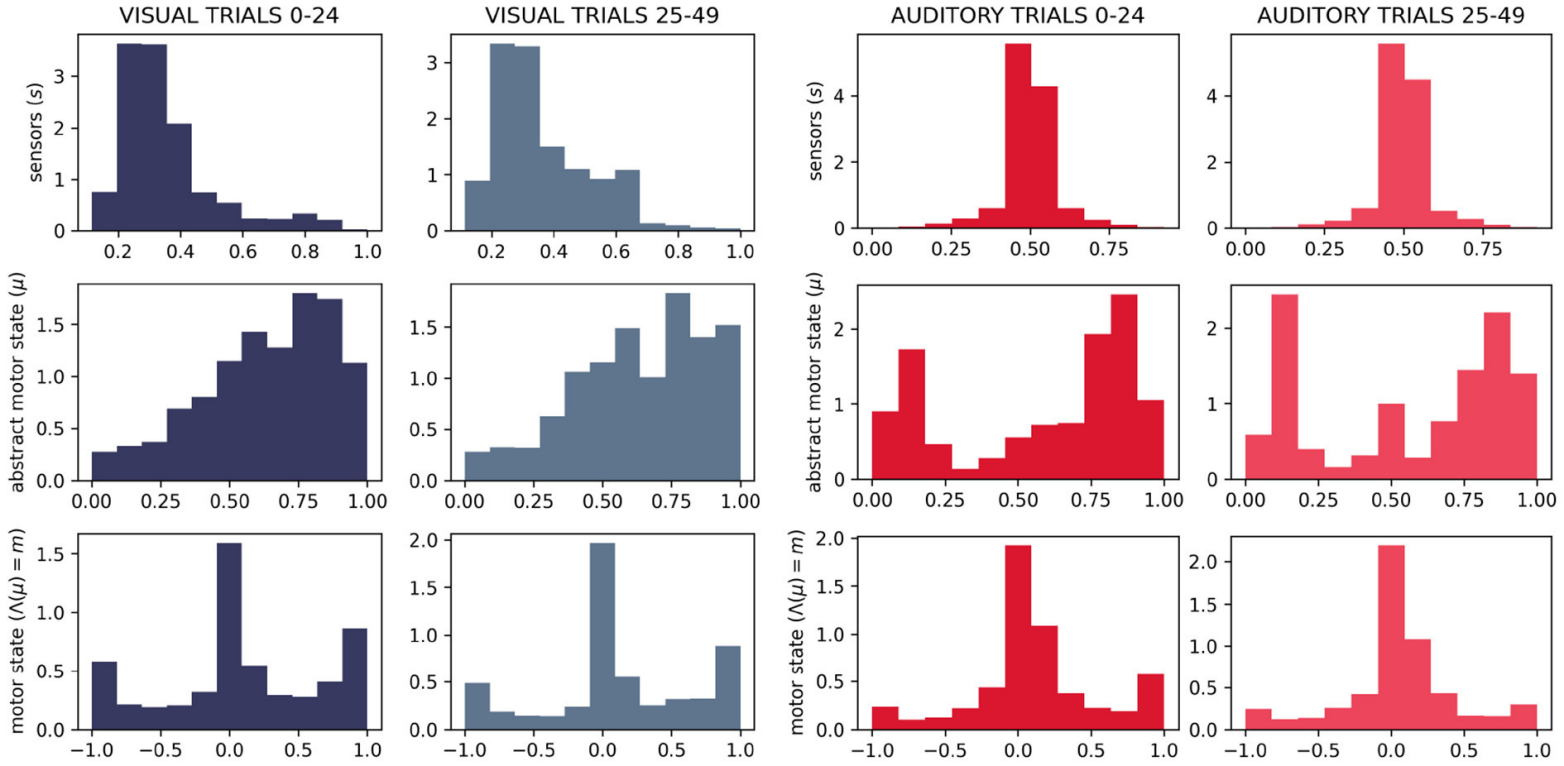
Histograms showing in-group similarity in sensorimotor state for visual trials **(left)** and auditory trials **(right)**.

We do, in fact, see differences in the distributions of motor states between the two sensory conditions (Figure 11). Visually, we can see that the distributions of motor (and abstract motor) states in the visual robots seem different than that of the auditory robots (Figure 11); and the distributions of motor states within these groups in independent runs of the visual trial seem more similar to each other (Figure 12) than they are to the other group. We confirmed the statistical significance of these differences by using Kullback–Leibler divergence to assess the distributional conformity of 50 visual trials and 50 auditory trials. The within-group (intra-modal) distributions were significantly more similar than the between-group (inter-modal) distributions for *m* (*t* = –4.67, *p* = 0.0114) and for μ (*t* = –8.507, *p* = 0.0215).

In the absence of the self-reinforcing dynamic of the IDSM, habits, motor activity would be random, in which case we would expect the same distribution of motor states in both sensory modalities. The histograms in (new) (Figures 11, 12) show that this is not the case, and thus provide support for the claim that the sensorimotor contingencies implicit in the different sensory modalities constrain not only the sensory dynamics, but also motor dynamics—and in doing so, they play an important role of constraining the form of emergent autonomous sensorimotor dynamics.

**Figure 13:**
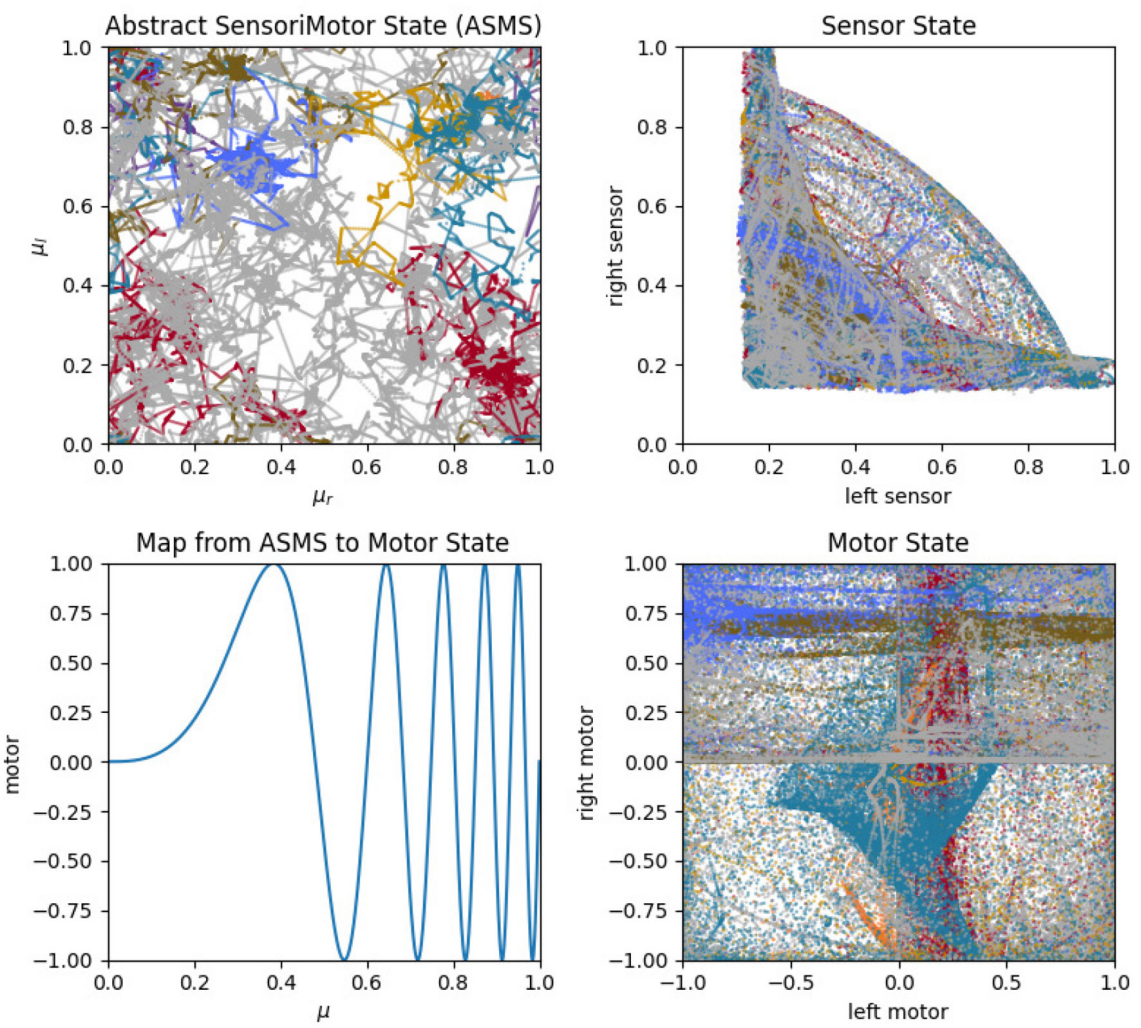
Sensorimotor dynamics for the visual robot. These plots show motor trajectories in abstract sensorimotor space **(upper left)**; two projections of the actual sensorimotor trajectories of the robot **(upper** and **lower right)**; and the mapping function, Λ, that transforms the abstract motor state to an actual motor state (see Section 3.4.2). Colors indicate habits as in previous plots.

**Figure 14:**
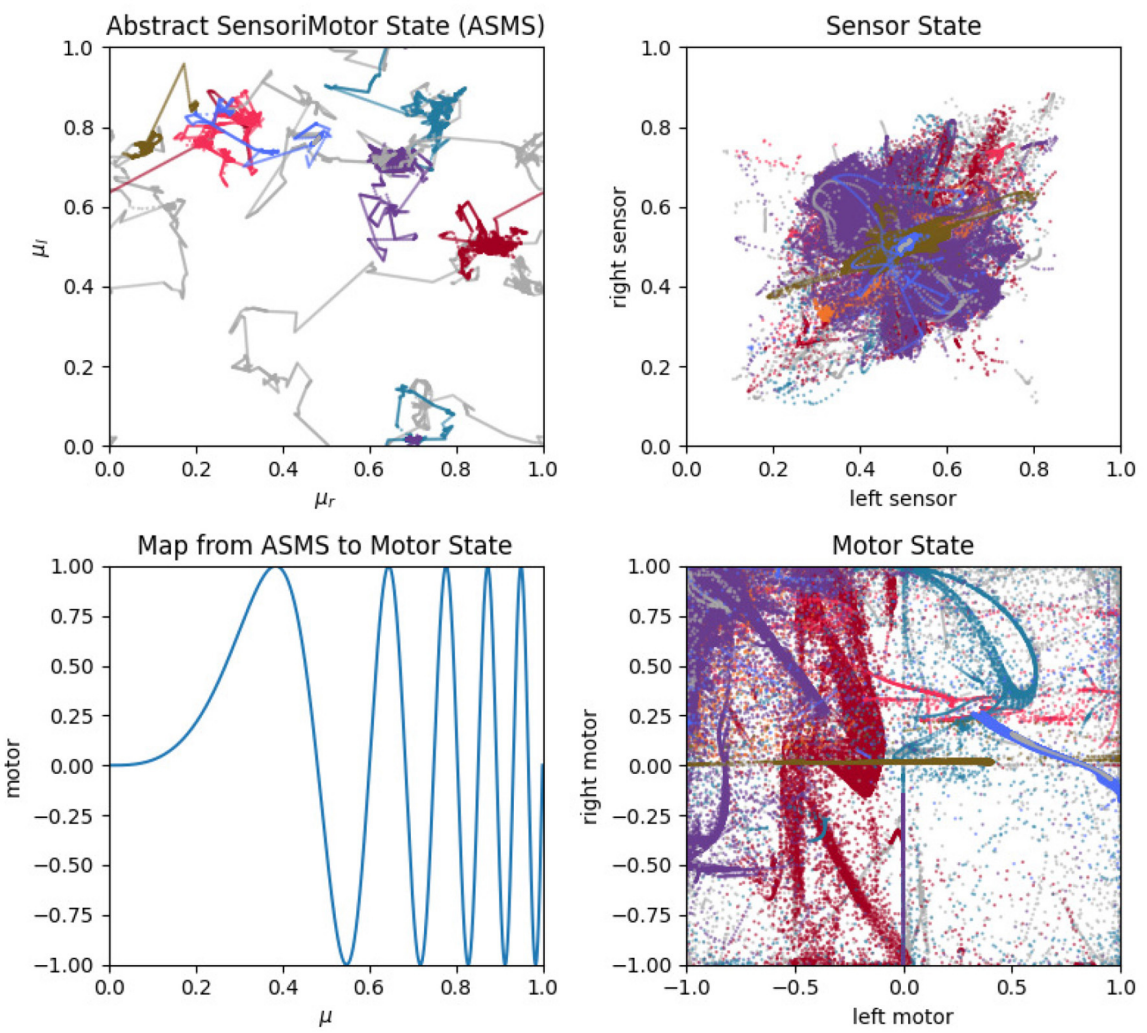
Sensorimotor dynamics for the auditory robot. This figure is the same as Figure 13 but plotted for the auditory robot.

The primary point that we wish to communicate using this model is that sensorimotor contingencies influence which patterns of autonomous sensorimotor behavior can emerge and persist. It is worth emphasizing that the plots in Figures 10, 13, 14 are not plots of the sensorimotor contingencies of the robot—they are plots of the robots' self-maintaining habits. Nevertheless, we see in these plots the *influence* of sensorimotor contingencies—the way that the contingencies have constrained the set of habits that can form and self-stabilize, producing the regular patterns described in the paragraphs above.

The two different sensory modalities entail two different sets of sensorimotor contingencies. The different sensorimotor contingencies each imply a different set of possible self-stabilizing habits, and so the habits that form with one sensory modality are more similar to each other than they are to the habits that form with the other sensory modality. Two *visual* habits will be different from each other, but they will generally be more similar to each other than auditory habits. This is what we mean by the claim that the form of sensorimotor habits are constrained by their sensory modality and it is apparent in the regularities described above and seen in Figures 13, 14. In this account, sensorimotor contingencies play an important role. They influence the form of the emergent autonomous sensorimotor strutures, but the rules themselves are not internalized or learned in any other way.

## 5. Discussion

### 5.1. Solving somebody else's problems vs. having your own problems

The robots we have just presented display a number of interesting and challenging features for traditional problem-solving centered artificial intelligence (biologically inspired or otherwise). We see in this model how sensorimotor dynamics can have problems of their own. As we have seen AI and robots more specifically are generally built to solve problems. But whose problems? Perhaps the engineers' problems or those of their clients, perhaps animal problems that require robotic models to be better understood, maybe societies problems but certainly not those of the robot itself. It is thus important to distinguish between having one's problem vs. being able to solve a problem. A bacterium that swims up a gradient toward the resources it needs to survive is doing both. It has a problem (it needs food to persist) and it solves that problem by navigating through its environment. AI, on the other hand, is capable only of the former. A “self-driving” car that takes me to a restaurant dinner is solving a problem (my need for food), but that problem is mine, and not the car's.

The enactive robots presented in this paper where not designed to do anything, to solve any problem. None of our analysis was described in terms of success or failure at the performance of a task. The architecture makes habits emerge and with them a basic sense of having a problem: the habit *needs* to enact itself in order to persist. The norms of behavior here are thus only in terms of the persistence of the behavior itself (and concurrently the mechanism that produces it). Surely, life is full of problems, we certainly have enough with our own and don't need to be concerned with creating new ones …unless we want to understand what it is to have a problem and how life is, itself, a source of problems. Enactivism embraces a deep conception of life as the self maintenance of a network of precarious processes. In this sense life is inherently a source of problems. By making it possible for habits to emerge and sustain their own existence through recurrent sensorimotor interaction we have shown how this conception of life can be practically transferred to a “way of life” in sensorimotor robotics.

A research program on enactive robotics can and must address how sensorimotor life is a source of problems. We can move beyond the single habit and envision how the network of habits that constitutes the agents can display emergent problems of its own: that of keeping the whole network alive, coping with variations on the way the environment affords or precludes to enact them. This line of enquiry requires further work but the present model requires little development to start addressing it.

### 5.2. Constraints and form in biological and behavioral explanation

Biological inspiration in robotics can also move beyond the analogy between metabolic or physiological life and its sensorimotor counterpart. In particular the models presented and analyzed in this paper show how sensorimotor life and the problems that emerge within are constrained by sensory embodiment and how such constraints constitute an important part of what it means to explain life.

Whether it is understood as more fundamental-than, complementary-to or directly at odds-with (Newman, 2018) natural selection, it is the repeated, regular, robust and often phylogenetically independent appearance of forms or structures in biology what becomes in itself an object of study that cannot be reduced-to or deduced-from life as a response to environmental problems. In this sense biologically inspired enactive robotics can learn from biological development and its organization and import into psychology some explanatory strategies followed by biologists. Darcy Thompson, René Thom, Pere Albert, etc. all conceived biological forms to be both *explananda* and *explanans* on themselves. Homologies, analogies, inherency, are all concepts directed to capture the re-emergence of certain forms (and functions!) in biological organisms. The notion of constraint plays here a fundamental role. The way in which different layers of materiality and self-organization limit and channel the emergence of viable forms is essential to biological explanation.

If biological inspiration within the problem-solving paradigm concluded that the material embodiment of robots permits to offload and transform the problems to be solved, the biological inspiration of the enactive paradigm can conclude that the embodiment loads and informs the problems that constitute the sensorimotor agent. As we have seen, sensory modalities constraint motor trajectories, which in turn, shape how habits get stabilized. Thus, in a way that parallels the explanatory role of constrains in evolution, we can hypothesize that behavioral variability is not free (to explore potential solutions to cognitive problems) but is constrained and channeled by the embodiment of the agent.

Now, before the advent and widespread influence of evolutionary theory in biology, the latter synthesis with molecular biology and subsequent expansion to psychology, sociology and even epistemology, or philosophy more generally, the concept that was key for the continuity between life and mind was that of habit.

### 5.3. Enactive robotics revisited

There are different takes on how enactivism translates into robotics. As mentioned in the introduction it is possible to simply reject representational functionalism, or to claim for the importance of embodiment, or to demand that robot be endowed with living bodies, or to introduce some feedback mechanisms that parallel those provided by emotion bearing bodies. We have taken a different approach—that envisioned by Smithers (1997) and latter developed by Di Paolo (2003), Barandiaran and Moreno (2006), and Barandiaran (2008). What a research programme in enactive robotics entails is the study of the organization of sensorimotor life: the form of viability of different habits, the topology of the network of habits that unfolds over development, the shape of the habitat that is thus constructed, the structure of the world that is experienced. Habit-based robots as designed here are capable of individuating habits and of creating an ecology of habits that can easily be understood as a form of sensorimotor self.

Not only has enactivism informed robotics (Ziemke and Lowe, 2009; Vernon, 2010) but robotics has often served enactivism (Beer, 1995; Di Paolo, 2003; Aguilera et al., 2016) by clarifying its claims, pushing theoretical development, operationalizing its concepts or penetrating diverse problems. The model we developed here can be aligned with the latter. It can be used to clarify and make explicit the often obscure original formulation of enactivism that “cognition in its most encompassing sense consists in the enactment or bringing forth of a world by a viable history of structural coupling” (Varela et al., 1991, p. 205). We have shown how a robot endowed with an IDSM can bring into being a number of habits, the world that it brings forth is the habitat, more specifically, the structured set of sensorimotor contingencies that the agent inhabits or enacts. This habitat must be viable in the sense that habits must be sustainable and results from a (developmental) history of sensorimotor (structural) coupling. And in so doing, we have demonstrated how sensorimotor contingencies can *directly* constrain or “sculpt” the form taken by sensorimotor habits without requiring any virtualization, i.e., without any internal model or representation of the sensorimotor contingency.

Perhaps it is worth clarifying why the nodes do not constitute an internal model or representation. The nodes do not stand-for something else other than the sensorimotor dynamics they partake in. It is not possible to operate upon the nodes in a decoupled offline mode that is not itself the enactment of a behavior, and there is not additional module or subsystem “consuming” such nodes to carry out any further operation. In these ways, the nodes do not represent a behavior or habit, but they embody it—they constitute the habit *together* with elements of the robot's bodily and environmental dynamics.

## 6. Conclusion

A central focus across the cognitive sciences is upon problem-solving ability and tremendous progress has been made in understanding how to mechanize problem solving. Much of AI and robotics research is validated by how well some artifact (neural network, human being, robot, etc.) performs at a problem-solving task (chess, maze navigation, bipedal walking, etc.).

However, the conflation of “problem-solving ability” with all of the phenomena associated with ‘being a mindful body leaves out a number of features that demand be put at the center of (enactive) theorizing: historicity, embodiment, habitat, precariousness, identity, norm-establishing, etc. All these dimensions of mental life are worthy of study and remain outside of the problem-solving frame that scaffolded the development of Artificial Intelligence and Robotics.

We have here presented a set of robots with different sensory modalities that spontaneously develops a complex ecology of sensorimotor habits. These are constrained by the sensory modality of the robot and give rise to sensorimotor habitats of specific forms. The very nature of habits thus developed, understood as self-sustaining forms of sensorimotor activity, has shown how robots must first have their own problems instead of solving those posited by external observers; and, that, in doing so, they must assert a form of life whose structure and topology must be taken as the object of study. In particular we have seen how the form of sensory embodiment shapes potential sensorimotor contingencies and these constraint the shape and type of sensorimotor habits that emerge during development.

To be alive is not a computable function but the way in which materiality (implementation), behavior and function are deeply intertwined. What was once claimed as the triumph of functionalism as the clear conceptual separation between matter, behavior and machine state transitions is now its deepest weakness. The enactive approach brings all three together again. To be fair it is not the materiality of the robot's “body” that is a stake here (neither does the simulation itself possess any body beyond the computer in which the simulation was carried out, not the physical body, e.g. wheels and sensors, would be at stake was the robot to be implemented in real life), but the materiality of the sensorimotor mapping, its precarious existence, its fading structural stability.

Perhaps the “artificial sciences” (AI, artificial life, robotics, exploratory computational modeling, etc.) would benefit from similarly investing more time in targets other than problem-solving ability. Biologists are sometimes accused of suffering “physics envy”—i.e., wishing that the objects of their study were more easily and completely summarized by simple, provable equations. Perhaps we enactivist and embodied researchers can be accused of “problem-solving envy” a desire for our artifacts and theories to be equally or more capable of solving problems as the expert systems or disembodied neural networks of other problem-solving focused approaches. And perhaps this envy is a distraction, and impeding our progress toward understanding minds. In fact, robotics cannot only reveal itself as an engineering practice directed at solving problems but also as a philosophical practice aimed at posing the right problems. This is a contribution that enactive robotics is ready to do.

## 7. Permission to reuse and copyright

Figures, tables, and images will be published under a Creative Commons CC-BY license and permission must be obtained for use of copyrighted material from other sources (including re-published/adapted/modified/partial figures and images from the internet). It is the responsibility of the authors to acquire the licenses, to follow any citation instructions requested by third-party rights holders, and cover any supplementary charges.

## Data availability statement

The raw data supporting the conclusions of this article will be made available by the authors, without undue reservation.

## Author contributions

ME conceived, implemented, ran simulations and experiments, and authored the manuscript. XB contextualized research, interpreted results, and authored the manuscript. Both authors contributed to the article and approved the submitted version.

## Figures and Tables

**Figure A1:**
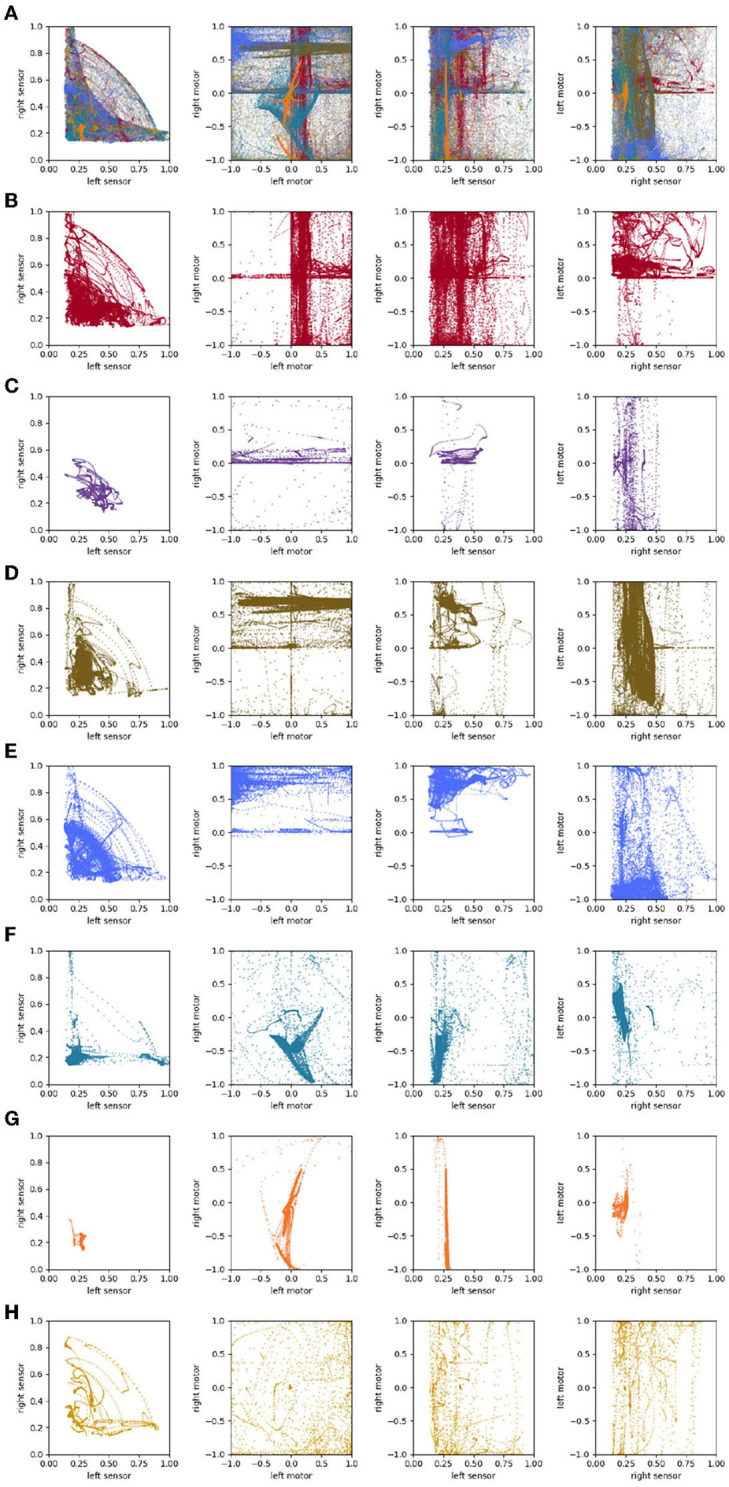
Projections of the sensorimotor trajectories of the visual robot. Colors and letters **(B–H)** are included to facilitate comparison with Figure 3. **(A)** Shows the superposition of trials **(B–H)**.

**Figure A2:**
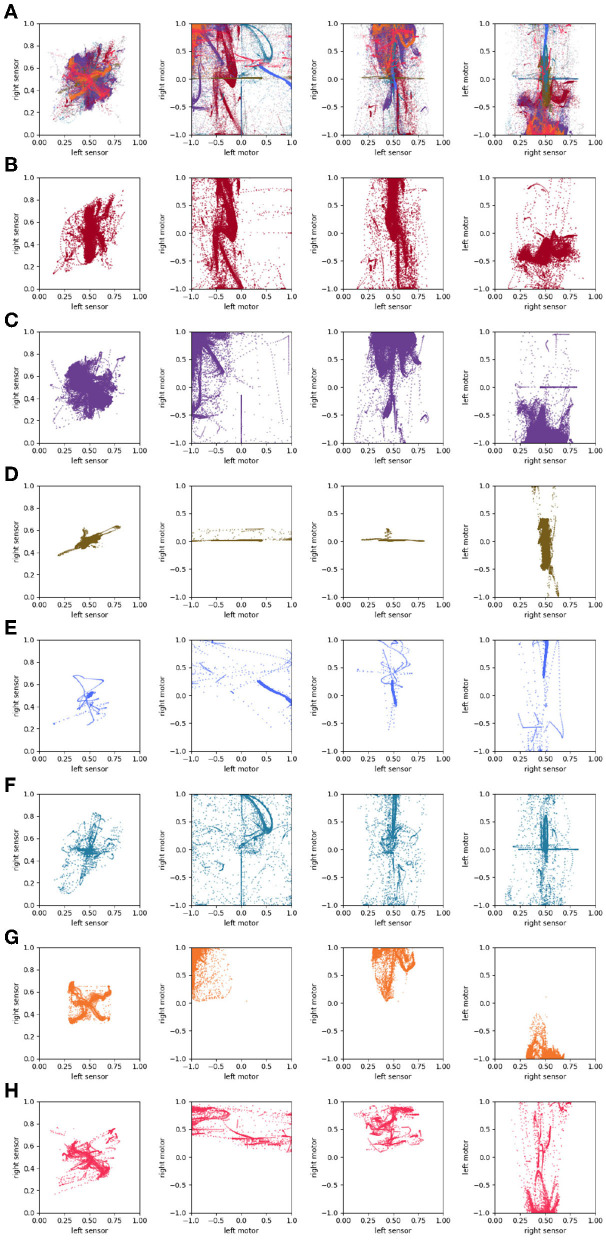
Projections of the sensorimotor trajectories of the visual robot. Colors and letters **(B–H)** are included to facilitate comparison with Figure 7. **(A)** Shows the superposition of trials **(B–H)**.
